# The p38 mitogen activated protein kinase inhibitor losmapimod in chronic obstructive pulmonary disease patients with systemic inflammation, stratified by fibrinogen: A randomised double-blind placebo-controlled trial

**DOI:** 10.1371/journal.pone.0194197

**Published:** 2018-03-22

**Authors:** Marie Fisk, Joseph Cheriyan, Divya Mohan, Julia Forman, Kaisa M. Mäki-Petäjä, Carmel M. McEniery, Jonathan Fuld, James H. F. Rudd, Nicholas S. Hopkinson, David A. Lomas, John R. Cockcroft, Ruth Tal-Singer, Michael I. Polkey, Ian B. Wilkinson

**Affiliations:** 1 Department of Experimental Medicine and Immunotherapeutics, University of Cambridge, Cambridge, United Kingdom; 2 Cambridge Clinical Trials Unit, Cambridge University Hospitals NHS Foundation Trust, Cambridge, United Kingdom; 3 NIHR Respiratory Biomedical Research Unit, Royal Brompton & Harefield NHS Foundation Trust and Imperial College, London, United Kingdom; 4 GSK R&D, King of Prussia, Pennsylvania, United States of America; 5 Department of Respiratory Medicine, University of Cambridge & Cambridge University Hospitals NHS Foundation Trust, Cambridge, United Kingdom; 6 Department of Cardiovascular Medicine, University of Cambridge & Cambridge University Hospitals NHS Foundation Trust, Cambridge, United Kingdom; 7 Department of UCL Respiratory, Division of Medicine, Rayne Building, University College London, London, United Kingdom; 8 Department of Cardiology, Wales Heart Research Institute, Cardiff University, Cardiff, United Kingdom; Pulmonary Research Institute at LungClinic Grosshansdorf, GERMANY

## Abstract

**Background:**

Cardiovascular disease is a major cause of morbidity and mortality in COPD patients. Systemic inflammation associated with COPD, is often hypothesised as a causal factor. p38 mitogen-activated protein kinases play a key role in the inflammatory pathogenesis of COPD and atherosclerosis.

**Objectives:**

This study sought to evaluate the effects of losmapimod, a p38 mitogen-activated protein kinase (MAPK) inhibitor, on vascular inflammation and endothelial function in chronic obstructive pulmonary disease (COPD) patients with systemic inflammation (defined by plasma fibrinogen >2·8g/l).

**Methods:**

This was a randomised, double-blind, placebo-controlled, Phase II trial that recruited COPD patients with plasma fibrinogen >2.8g/l. Participants were randomly assigned by an online program to losmapimod 7·5mg or placebo tablets twice daily for 16 weeks. Pre- and post-dose ^18^F-Fluorodeoxyglucose positron emission tomography co-registered with computed tomography (FDG PET/CT) imaging of the aorta and carotid arteries was performed to quantify arterial inflammation, defined by the tissue-to-blood ratio (TBR) from scan images. Endothelial function was assessed by brachial artery flow-mediated dilatation (FMD).

**Results:**

We screened 160 patients, of whom, 36 and 37 were randomised to losmapimod or placebo. The treatment effect of losmapimod compared to placebo was not significant, at -0·05 for TBR (95% CI: -0·17, 0·07), p = 0·42, and +0·40% for FMD (95% CI: -1·66, 2·47), p = 0·70. The frequency of adverse events reported was similar in both treatment groups.

**Conclusions:**

In this plasma fibrinogen-enriched study, losmapimod had no effect on arterial inflammation and endothelial function at 16 weeks of treatment, although it was well tolerated with no significant safety concerns. These findings do not support the concept that losmapimod is an effective treatment for the adverse cardiovascular manifestations of COPD.

## Introduction

Chronic Obstructive Pulmonary Disease (COPD) is a complex condition, which is associated with extra-pulmonary manifestations, such as systemic inflammation and cardiovascular disease, that increase patient morbidity and mortality [1]. Current pharmacological treatments for COPD are generally limited to inhaled bronchodilators and corticosteroids, which do not address extra-pulmonary features. Diversifying therapeutic options to focus on systemic manifestations, and by implication more accurate stratification of COPD patients for such treatments, might improve clinical outcomes.

p38 mitogen-activated protein kinases (MAPK) are signalling molecules that regulate cellular responses to extra-cellular stresses [2]. They also regulate pro-inflammatory cytokines, and have a key role in the initiation and development of inflammatory diseases, including COPD and atherosclerosis [2] [3]. Therefore, pharmacological inhibition of the p38 MAPK pathway, may represent a potential novel approach to treating the adverse cardiovascular manifestations of COPD. To date, losmapimod (GW856553 Brentford UK), a selective p38α/β MAPK inhibitor has been evaluated as an anti-inflammatory therapy in different patient populations including COPD and cardiovascular disease. In COPD, losmapimod reduces acute phase circulating proteins such as fibrinogen, and a reduction in hyperinflation have been described [4]. In cardiovascular disease, losmapimod attenuated arterial inflammation in atherosclerosis [5], improved endothelial function in hypercholesterolemic subjects [6], and showed a trend towards improved left ventricular function following non-ST elevation myocardial infarction [7].

Arterial inflammation, measured by positron emission tomography (PET) imaging, is a promising vascular biomarker, which has been shown to improve prediction of cardiovascular events beyond traditional risk factors, and also informed on the timing of such events [8]. Brachial artery flow-mediated dilatation (FMD) is a measure of endothelial function which is associated with cardiovascular outcomes, and has prognostic value in different clinical cohorts [9]. COPD patients have increased arterial inflammation compared to healthy controls, regardless of smoking history [10], and also demonstrate impaired endothelial function [11]. Additionally, systemic inflammation is hypothesised to be a causal factor in the increased cardiovascular risk observed in COPD patients [1]. Plasma fibrinogen, a stable biomarker of systemic inflammation in COPD, is positively associated with cardiovascular risk in the general population, and is implicated in the development of vascular dysfunction and atherosclerosis [12]. It has also recently been qualified by the Food and Drug Administration (FDA) as a drug development tool for predicting the risk for COPD exacerbations, and mortality in COPD [13].

The EVOLUTION (Evaluation of Losmapimod in Chronic Obstructive Pulmonary Disease patients with systemic inflammation stratified by fibrinogen) trial, was an experimental medicine study, designed to test the hypothesis that losmapimod, as an anti-inflammatory agent, reduces arterial inflammation and concomitantly improves endothelial function (our co-primary endpoints of the study) in a cohort of COPD patients with evidence of systemic inflammation, stratified by plasma fibrinogen. Secondary endpoints included safety and tolerability of losmapimod, and the effects of losmapimod on arterial stiffness and blood biomarkers of inflammation.

## Materials and methods

The EVOLUTION study design and protocol have been previously published [14] and the protocol (S1 Protocol) is included in the Supporting Information. Key trial information is summarised below.

### Study design

EVOLUTION was a randomised, double-blind placebo-controlled, parallel-group, stratified experimental medicine trial, which recruited from two UK tertiary centres (Addenbrooke’s Hospital, Cambridge and the Royal Brompton Hospital, London). The study was approved by Cambridge South Research Ethics Committee and was registered with ClinicalTrials.gov (NCT01541852). It was conducted in accordance with the Declaration of Helsinki and Good Clinical Practice standards, and written informed consent was obtained from all patients. The study was active from 1^st^ April 2012 when the first patient was recruited till the 6^th^August 2014 when the final patient recruited completed the study. Based on sample size calculation, the planned recruitment target of the trial was met.

### Participants

**Fig 1:**
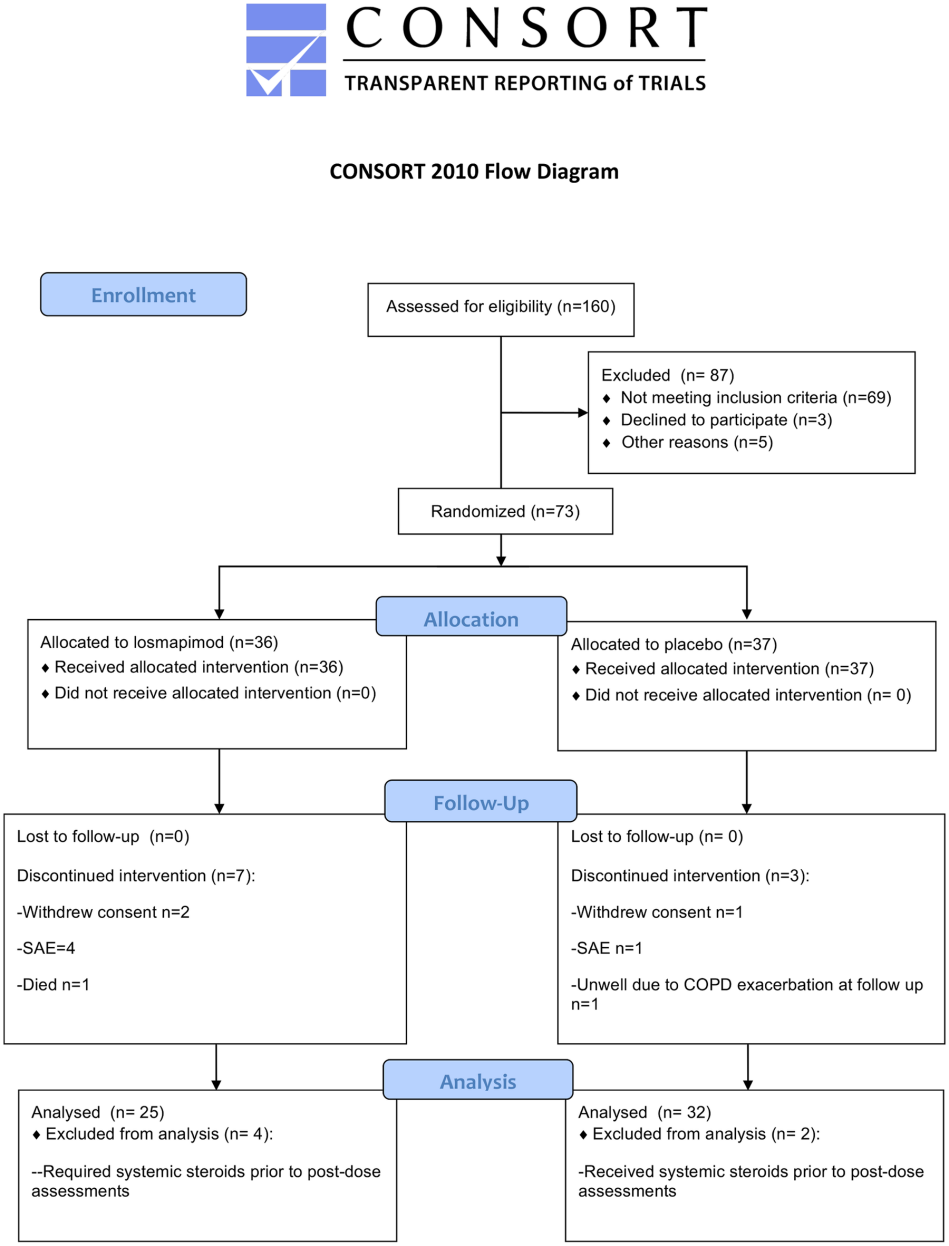
Consort diagram of EVOLUTION trial. (*expanded in S3 Table).

Eligible patients were aged 50–85 years, with a known diagnosis of COPD (confirmed by post-bronchodilator spirometry Forced Expiratory Lung Volume in 1 second/Forced Vital Capacity, (FEV_1_/FVC ratio <0·70)), or with spirometry which fulfilled GOLD-Unclassified (GOLD-U: FEV_1_/FVC ≥0·7 and FEV_1_ <80% predicted) categorisation [15]. However, no GOLD-U patients were recruited to the study. All patients were current or ex-smokers with at least a 10-year pack year history and a plasma fibrinogen >2·8g/L. This level of plasma fibrinogen was selected as the stratification threshold for entry to the study because although there is no evidence of a fibrinogen threshold that stratifies for cardiovascular risk [16], values greater than 2.7g/L are associated with an increased risk of hospitalisation, and worse FEV_1_ [17], (and poor lung function defined by FEV_1_, is similarly associated with increased cardiovascular risk [18]). All patients maintained on their standard-of-care medications prior to and during the study, including prescribed medications for cardiovascular comorbidity and COPD. Full inclusion and exclusion criteria of the trial including information regarding amendment to the trial inclusion criteria, are reported in S1 and S2 Tables respectively, and a study flow-chart of visits and assessments performed are shown in S1 Fig. The trial consort diagram is shown in Fig 1 with further information also provided in S3 Table. The consort checklist for the EVOLUTION trial is found in S1 Appendix.

### Randomisation and masking

Subjects were randomised (1:1) to receive either losmapimod 7.5mg or matching placebo twice daily for 16 weeks. An online coded program was used to randomise patients stratified by trial site. All trial personnel, including investigators giving trial interventions, assessing outcomes, and analysis of data, and patients were blinded throughout the study to treatment allocation. Study medication was provided by GSK.

### Assessments for primary endpoint

#### Vascular PET/CT imaging

**Fig 2:**
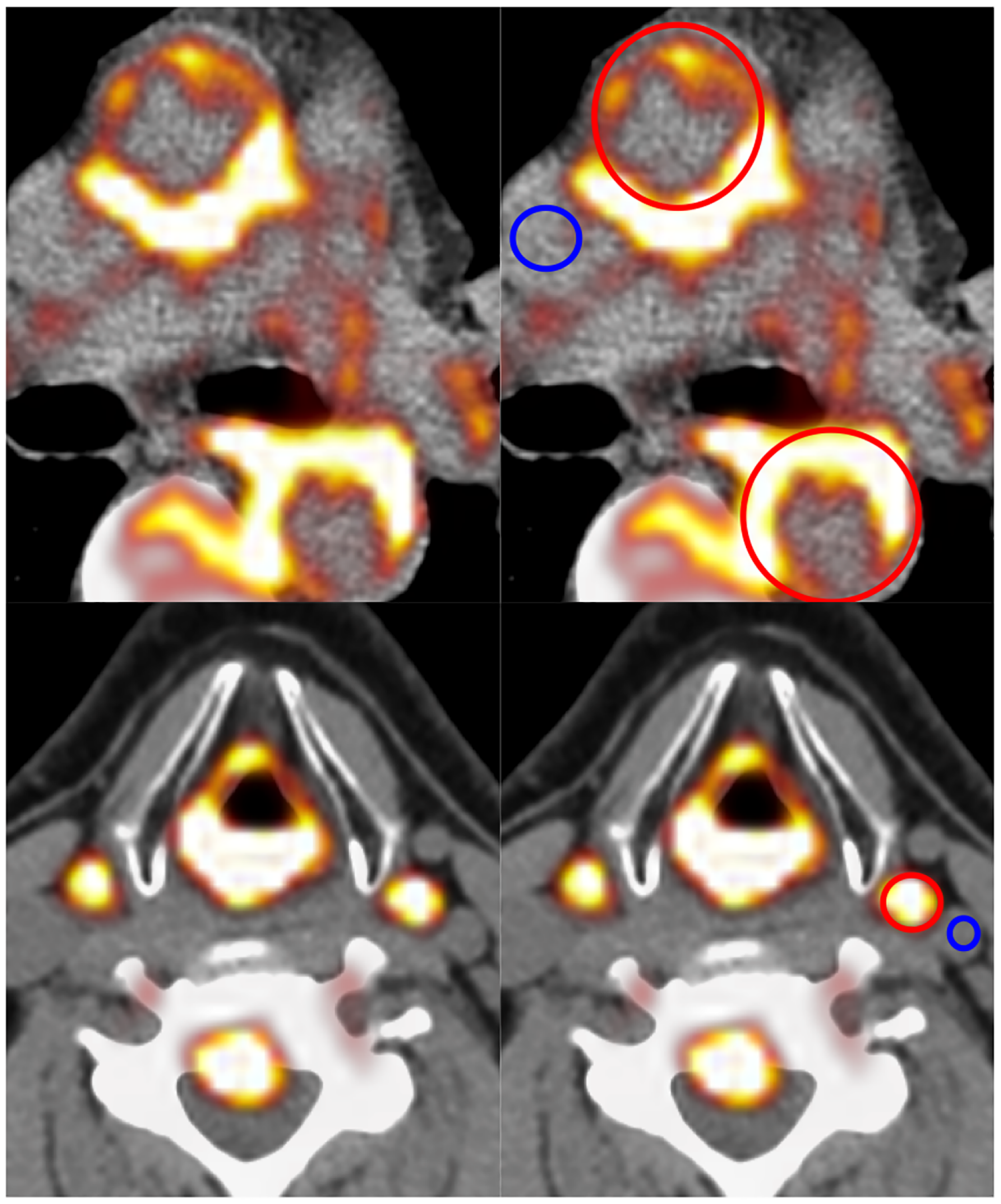
Calculation of arterial tissue-to-blood ratio (TBR) as a measure of arterial inflammation. Calculation of TBR: ratio of FDG tracer concentration in the artery divided by FDG concentration in the venous blood pool (superior vena cava for aorta, internal jugular for carotid).

^18^F-Fluorodeoxyglucose PET paired with attenuated corrected computed tomography (FDG PET/CT) imaging of the aorta and carotid arteries was performed before, and at 16 weeks of treatment, to evaluate tracer uptake as a surrogate measure of arterial inflammation. Further detailed information regarding imaging is explained in S1 Methods. For image analysis, Osirix open-source DICOM software v5·6 (Geneva, Switzerland) was used. A region of interest (ROI) including arterial wall and lumen was drawn on each axial slice of artery on the co-registered PET/CT scan and the maximum Standardised Uptake Value (SUVmax) recorded. Subsequently, each ROI was normalised by blood FDG concentration in the superior vena cava or jugular vein (for carotids), to yield an arterial maximum tissue-to-blood ratio (TBR) as a quantitative measure of arterial tracer uptake (Fig 2). Slices with a TBR >1·6, deemed ‘active slices’ were included in analyses.

The arteries were analysed as six arterial territories: the ascending, descending, abdominal aorta and right and left carotid arteries, and the aortic arch. For each arterial territory and each scan, the average TBR was calculated by taking the mean of the TBR across all of the slices in the territory with a TBR >1.6. This produced the TBR of each arterial territory, for each scan. Change in TBR was pre-defined as the change in average TBR, from post- to pre-dose scans.

The “index vessel” (defined as the arterial territory with the highest average TBR at baseline) was selected as the *a priori* focus of interest to determine change over time with treatment or placebo. Arterial territories that could be the index vessel included the ascending, descending, abdominal aorta or either carotid artery, but not the aortic arch because higher inter-observer variability in analysing this region was previously reported. [19] Additionally, we report the change in TBR in each arterial region individually, including the aortic arch. All scans were analysed by an experienced reader, anonymised to patient identifiable information, treatment group, and visit number.

#### Endothelial function

Endothelial function was assessed by brachial artery FMD pre- and post-treatment using established ultrasonography techniques [20]. FMD was defined as the maximum percentage increase in artery diameter, after release of a blood pressure cuff inflated to supra-systolic pressure for 5 minutes to induce ischemia distal to the site of measurement (reactive hyperemia). After a 10-minute break, endothelial-independent dilatation was then assessed. This is the maximum percentage increase in artery diameter after administration of 25 μg of sublingual glyceryl trinitrate (GTN).

### Assessments for secondary endpoints

#### Arterial stiffness

Assessments were performed pre- and post-treatment. After a 15-minute rest, seated brachial blood pressure was recorded (OMRON-750CP, Omron Corp, Japan). Radial artery waveforms were then obtained using a high-fidelity micromanometer (SPC-301, Millar Instruments, Texas, USA) and recorded using Sphygmocor software (AtCor Medical, Sydney, Australia), which generated a corresponding central waveform and aortic augmentation index using the software’s validated transfer function. Augmentation index is a composite measure of arterial wave reflections. Supine carotid-femoral aortic pulse wave velocity (aPWV), which is a measure of aortic stiffness, was also measured using the same device, as previously described [21].

#### Systemic inflammation

Blood samples were collected at days 0, 14, 28, 56, 84 and 112 (16 weeks) of treatment and 2-weeks following cessation of treatment, for measurement of plasma fibrinogen (Klauss method) [22], and high-sensitivity C reactive protein (hsCRP) using a clinically validated laboratory assay.

#### Safety

Adverse events, safety laboratory parameters and haemodynamic variables were assessed throughout the study and electrocardiograms (ECGs) were performed pre- and at 16 weeks of treatment.

### Statistical analysis

This was an exploratory trial utilising a sample size calculation similar to our prior PET imaging trial of losmapimod in atherosclerotic patients on stable statin therapy [5]. We estimated that 30 COPD patients per treatment arm would provide a 90% chance of detecting a difference of 15% change from baseline TBR when comparing losmapimod to placebo. To estimate the treatment effect with 95% confidence intervals (CI) of losmapimod compared to placebo, change from baseline TBR (or percentage change from baseline FMD, for our co-primary endpoint of endothelial function) was analysed using linear regression models, with the change from baseline as the dependent variable, treatment as the independent variable, and baseline value and treatment site as covariates. Individual regression models were used for the primary endpoints of index vessel TBR, each arterial territory TBR, and FMD (and GTN), and since change from baseline value was the dependent variable, only patients with both baseline and follow up data were included in analysis. For FMD and GTN analyses, pre-test mean brachial artery diameter was also included as a covariate in each regression model. Similarly, secondary endpoints were examined using the same regression analysis technique to assess treatment effect of losmapimod versus placebo. Statistical analysis was performed per protocol, and excluded patients who required systemic corticosteroids within four weeks of post-dose assessments. The reason for excluding these patients from primary endpoint analysis was concern that recent treatment with systemic corticosteroids would confound analysis of the treatment effects of losmapimod. Safety analysis included all randomised patients who had received at least one dose of study drug (losmapimod or placebo). Blood biomarker data were log transformed prior to analysis. *Post hoc* analysis was performed that excluded subjects who had a COPD exacerbation at anytime during the trial, where an exacerbation was defined as clinical deterioration in respiratory symptoms requiring treatment with antibiotics and/or systemic steroids. A *post hoc* analysis, which excluded patients with any known cardiovascular medical history at baseline (for example hypercholesterolemia, and therefore any patients on statins, or other cardiovascular medications), was performed. A p-value <0·05 was deemed significant for all statistical analyses. All data are presented as mean±standard deviation (SD), percentages, or with 95% confidence intervals (CI). Analyses were performed using R version 3·0·0 for Microsoft Windows with R-Studio version 0·98·953. Raw data of the trial are provided in S2 Appendix.

## Results

The flow of participants in the trial is shown in the consort diagram (Fig 1) and further information is provided in the Supporting Information supplement (S3 Table). 160 participants were assessed for eligibility at enrolment, of whom 73 were randomised to receive either losmapimod (n = 36) or placebo (n = 37). Demographics and baseline characteristics of patients are summarised in Table 1. The mean age of the whole trial cohort was 68±7years, 70% were male, 14% were current smokers, total pack years smoked were 47±25 years, FEV_1_ was 1·35±0·58 litres (50±21% predicted). Mean fibrinogen pre-dose was 3·52±0·63 g/L and hsCRP was 5.64±7·39 mg/L.

**Table 1:** Demographics of the trial cohort by treatment group as baseline.

Demographics	Losmapimod n = 36	Placebo n = 37
Age (years)	67±8	68±7
Male	25 (69%)	26 (70%)
Ex-smoker	32 (88%)	34 (91%)
Total pack years (n)	48±24	43±25
BMI (kg/m^2^)	26±4	26±4
**Cardiovascular medical history**		
CVD disease*	5 (14%)	3 (8%)
Hypertension	11 (31%)	7 (19%)
Hypercholesterolaemia	3 (8%)	4 (11%)
**Spirometry**		
FEV_1_ (L)	1·32±0·60	1·40±0·54
FEV_1_ percentage predicted (%)	50±19	52±22
FVC	2·93±0·90	3·12±0·80
FVC percentage predicted (%)	86±16	88±20
**Concomitant inhaled respiratory medication** †		
ICS and LABA (%)	27 (75%)	29 (80%)
ICS	2 (6%)	2 (6%)
LABA	2 (6%)	4 (11%)
LAMA	23 (63%)	27 (71%)
**Haemodynamic assessment**		
Systolic blood pressure (mmHg)	133±15	138±20
Diastolic blood pressure (mmHg)	77±10	80±10
Heart rate (bpm)	74±12	74±12
Augmentation Index (%)	28±9	25±10
Aortic pulse wave velocity (%)	9·47±3·13	10·54±2·48
**Systemic Inflammation**		
hsCRP (mg/l)	3·6 (2·1–4·9)	2·3 (1·2–7·5)
Fibrinogen (g/l)	3·5 (3·1, 3·9)	3·3 (3·1–3·7)
White cell count (x10^9^/L)	7·1 (5·7–8·3)	7·0 (5·7–8·1)
**Concomitant cardiovascular medication**		
Statins (%)	12 (33%)	7 (19%)
Beta-blockers (%)	4 (11%)	0 (0%)
Calcium-channel blockers (%)	8 (22%)	6 (16%)
ACE inhibitors/A2RB (%)	9 (25%)	7 (19%)
Anti-platelets (%)	8 (22%)	4 (11%)

Mean±SD, median (IQR), n (%) are presented.

*CVD: history of angina, myocardial infarction, acute coronary syndrome, stroke, or revascularisation procedure. FEV_1_: Forced Expiratory Lung Volume in 1 second. FVC: Forced vital capacity. hsCRP: High sensitivity C-reactive protein.

^†^ICS and LABA: Combined inhaled corticosteroid and long acting beta agonist, ICS: inhaled corticosteroid, LABA: Long acting beta agonist, LAMA: Long acting muscarinic antagonist. ACE: Angiotensin, A2RB: Angiotensin 2 receptor blockers.

### Primary endpoints

**Fig 3:**
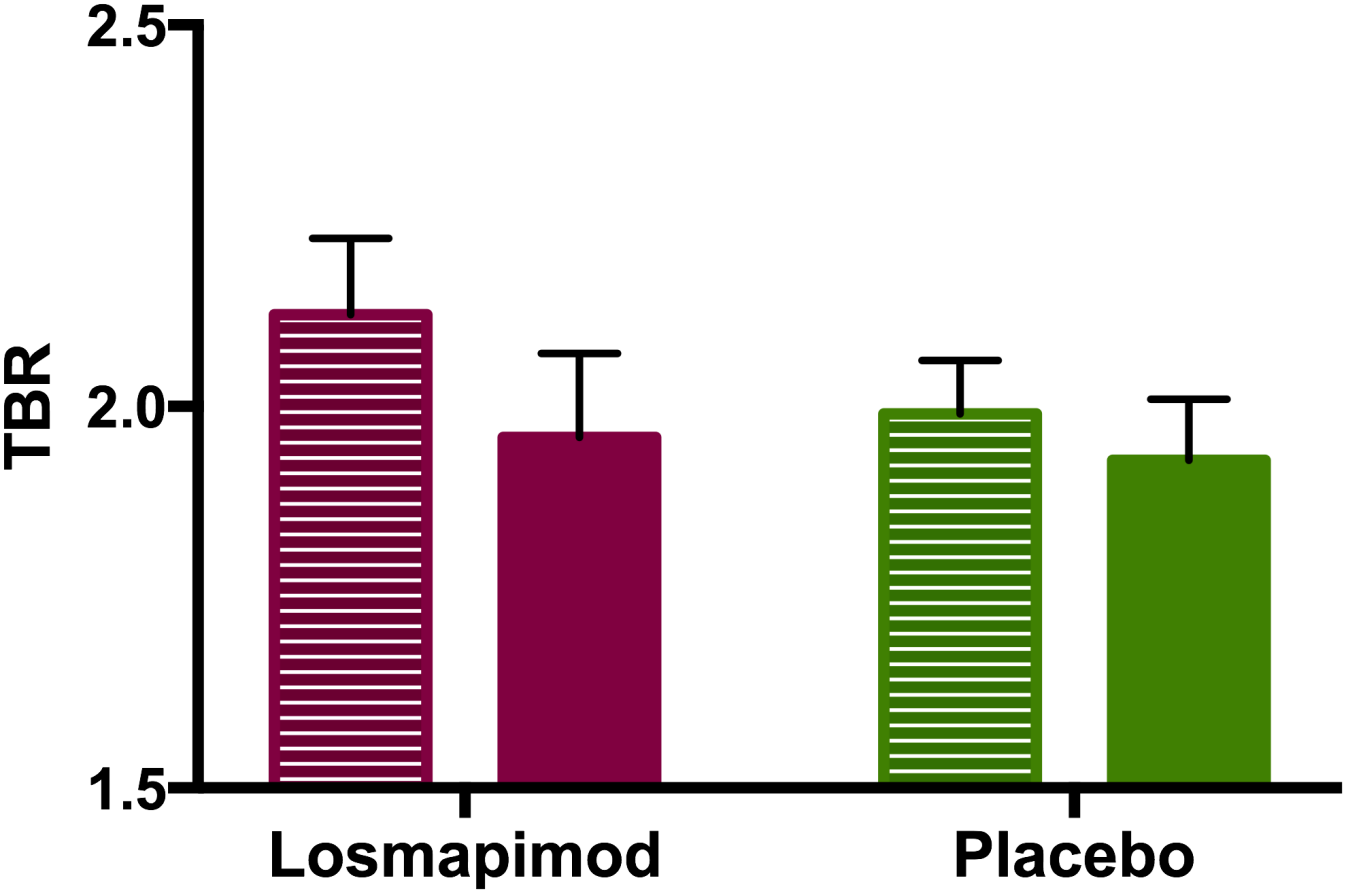
Tissue-to-blood ratio (TBR) at baseline and 16 weeks treatment with losmapimod or placebo in the index vessel. Losmapimod had no significant treatment effect, overall significance p = 0·42. Lined bars = baseline, solid bars at 16 weeks. Bars = mean values, error bars = 95% CI.

Losmapimod did not significantly reduce the primary imaging endpoint of change in TBR in the index vessel compared to placebo, (treatment effect of losmapimod on TBR: -0·05, (95% CI: -0·17, 0·07), p = 0·42; Fig 3). Table 2 summarises the average TBR of each anatomical arterial region pre- and post-dose, with point estimates of the treatment effect of losmapimod adjusted for placebo and site. S4 Table shows treatment effect data with coefficients of covariates from regression analyses also included. The aortic arch was the only anatomical region where there was a significant treatment effect of losmapimod on TBR compared to placebo.

**Table 2:** Tissue-to-blood ratio (TBR) for each arterial region at baseline and following treatment with losmapimod or placebo.

Vessel	Losmapimod		Placebo		Treatment Effect of Losmapimod	p-value of treatment effect
	Baseline	16 weeks	Baseline	16 weeks	Point Estimate (95% CI)	
	Mean±SD TBR		Mean±SD TBR			
**Index**	2·12±0·29	1·96±0·29	1·99±0·21	1·93±0·23	-0·05 (-0·17, 0·07)	0·42
**Arch**	2·10±0·28	1·95±0·23	1·92±0·20	1·93±0·19	-0·12 (-0·22, -0·03)	0·01
**Ascending**	1·96±0·26	1·90±0·26	1·85±0·18	1·86±0·20	-0·04 (-0·15, 0·06)	0·43
**Descending**	2·01±0·31	1·95±0·24	1·92±0·19	1·93±0·20	-0·04 (-0·14, 0·07)	0·49
**Abdominal**	1·99±0·22	1·92±0·24	1·88±0·20	1·90±0·20	-0·06 (-0·16, 0·03)	0·20
**R Carotid**	1·86±0·15	1·81±0·15	1·80±0·19	1·81±0·19	-0·05 (-0·13, 0·04)	0·29
**L Carotid**	1·86±0·20	1.80±0·19	1·80±0·16	1·88±0·27	-0·07 (-0·19, 0·07)	0·33

R Carotid = right carotid, L Carotid = left carotid. Mean±SD are presented. Baseline TBR and treatment site are covariates in linear regression models used to calculate the treatment effect of losmapimod versus placebo, on each artery listed.

**Fig 4:**
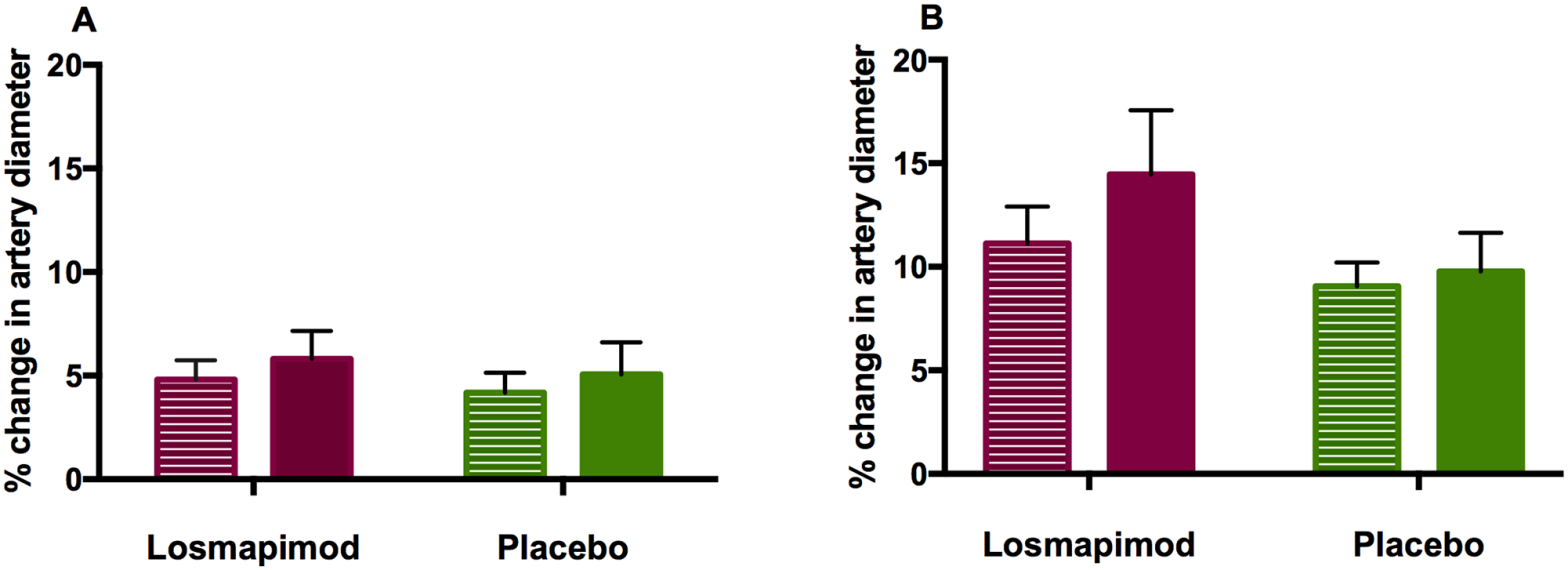
A) Percentage change in brachial artery diameter due to flow-mediated dilatation (FMD). B) Percentage change in brachial artery diameter following GTN administration, at baseline and 16 weeks treatment with losmapimod or placebo. Losmapimod had no significant effect on FMD compared to placebo, p = 0·70, although there was significant change (p = 0·03) following GTN administration. Lined bars = baseline, solid bars at 16 weeks. Bars = mean values, error bars = 95% CI.

For the co-primary endpoint of FMD, there was no significant treatment effect of losmapimod over placebo. The treatment effect of losmapimod did not change FMD significantly (+0·4%; 95% CI: -1·66, 2·47, p = 0·70). However, the effect of GTN was marginally higher (+3·25%; 95% CI: 0·41, 6·1, p = 0·03) in the losmapimod group compared to placebo, (Table 3 and Fig 4). Of note, there were no significant differences in either primary endpoint when stratified for study site. S4 Table shows treatment effect data with coefficients of covariates from regression analyses also included.

**Table 3:** Percentage change in brachial artery diameter with flow-mediated dilatation (FMD), and dilatation after glyceryl trinitrate (GTN) administration in the losmapimod and placebo groups of the study.

	Losmapimod		Placebo		Treatment effect of Losmapimod	p-value of treatment effect
	Baseline	16 weeks	Baseline	16 weeks	Point Estimate (95% CI)	
**FMD %**	4·80±2·56	5·80±3·48	4·17±2·75	5·06±4·04	0·40 (-1·66, 2·47)	0·70
**GTN %**	11·12±4·60	14·46±7·04	9·05±3·28	9·77±4·76	3·25 (0·41, 6·1)	0·03

Mean±SD are presented. Linear regression models with covariates of baseline percentage change, treatment sites and pre-test mean brachial artery diameter, were used to calculate treatment effect of losmapimod on FMD and GTN.

### Secondary endpoints

For the secondary endpoint of arterial stiffness, there were no statistically significant differences in augmentation index or aPWV following treatment in the losmapimod or placebo groups. The treatment effects of losmapimod were also non-significant for augmentation index (+0·83%; 95% CI: -4, 6%, p = 0·73), and aPWV (-0·01m/s; 95% CI: -0·95, 0·93 m/s, p = 0·98).

**Fig 5:**
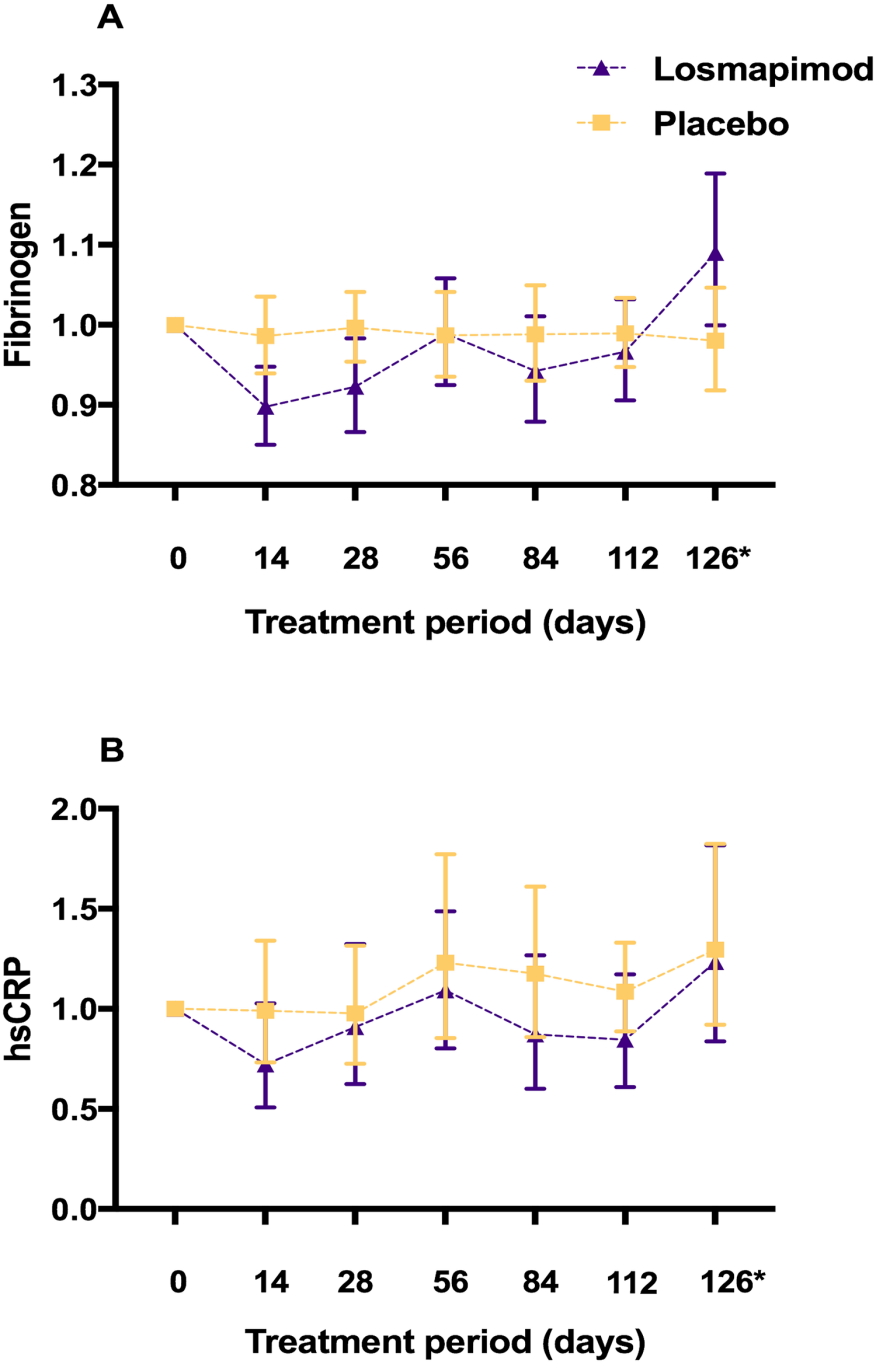
A) Plot of fibrinogen in the losmapimod and placebo treatment groups. Geometric mean of ratio to baseline value (95% CI) represented. *off drug B) Plot of hsCRP in losmapimod and control groups. Geometric mean of ratio to baseline value (95% CI) represented. *off drug.

We observed a significant decrease in mean fibrinogen in the losmapimod group (10% reduction compared to 0.3% in placebo group) on Day 14 of treatment (treatment effect -0·08; 95% CI: -0·15, -0·01, p = 0·03 corrected for baseline and placebo). However, there were no differences in fibrinogen levels from 4 weeks of treatment onwards, although there was a trend throughout the treatment period of the study, of greater attenuation in inflammatory biomarkers in the losmapimod group. A rebound of fibrinogen (albeit not hsCRP) above baseline, was observed two weeks after stopping losmapimod treatment. Fig 5 summarises the trajectory of fibrinogen and hsCRP values throughout the study.

Losmapimod was well tolerated in this COPD cohort. The frequency of adverse events reported was similar in the losmapimod and placebo groups (19 versus 12 due to COPD exacerbations, respectively, see S5 Table in the Supporting Information supplement). There were no clinically meaningful or statistically significant changes in laboratory parameters, vital signs, or ECGs over the course of the study in either treatment group. Serious adverse events (SAEs) were more frequent in the losmapimod treatment arm, and COPD exacerbations and pneumonia were the most common SAE reported (S4 Table).

### Post hoc analysis

Due to the high number of COPD exacerbations reported in this biomarker-enriched trial, a pre-specified *post hoc* analysis was performed to examine only stable patients (excluding patients who had a COPD exacerbation anytime during the trial). This showed a significant treatment effect of losmapimod compared to placebo on index vessel TBR (-0·13; 95% CI: -0·25, -0·02, p = 0·02). However, there was still no significant treatment effect on FMD (-0·22%; 95% CI: -2·36, 1·92, p = 0·83). Furthermore, *post hoc* analysis excluding patients with known cardiovascular history (and on cardiovascular medication), showed no significant treatment effect of losmapimod versus placebo, on index vessel TBR (+0·10; 95% CI: -0·15, 0·13, p = 0·91), or FMD (+0.55%; 95% CI: -2.71, 3.81, p = 0.73).

## Discussion

The primary finding of this experimental medicine study was that the anti-inflammatory p38 MAPK inhibitor, losmapimod, did not attenuate arterial inflammation nor improve endothelial function in systemically inflamed COPD patients, defined by a baseline fibrinogen >2·8g/l. To our knowledge, this is the first time fibrinogen has been used as a stratification biomarker for entry into a drug trial, and arterial inflammation used as a primary endpoint in an interventional COPD clinical trial.

Although our primary endpoint was negative, overall there was a trend for greater reduction in arterial inflammation with losmapimod treatment compared to placebo in all vessel territories, although this only reached significance in the aortic arch, and a larger study size may have highlighted treatment differences compared to placebo more clearly. Interestingly, in *post hoc* analysis of stable COPD patients who did not exacerbate during the trial, a significant treatment effect of losmapimod on the index vessel was observed. The respective 7% and 16% reduction in TBR observed in the aortic arch in the whole cohort, and index vessel in *post hoc* analysis when COPD exacerbators were excluded, may have clinical implications. For example intervention studies report a 6–15% reduction in TBR with other anti-inflammatory treatments such as anti-tumour necrosis-α therapy in rheumatoid arthritis patients, or high-dose statins in atherosclerosis [23] [24].

This COPD cohort had higher levels of systemic inflammation and index vessel TBR, than we previously reported in stable atherosclerotic patients [5]. However, the lack of significant treatment effect of losmapimod on vascular inflammation in this inflamed COPD cohort, is in contrast to our atherosclerosis data. We previously reported a treatment effect of -0·10 (95% CI: 0·19, -0·02; p = 0·01) on TBR after 12 weeks of losmapimod treatment in atherosclerosis patients already established on stable statin therapy [5]. Important differences between the two trials include the disease population targeted, and duration of therapy (16 weeks in our current trial versus 12 weeks previously). However, given the significant *post hoc* results when COPD exacerbators were excluded, a possible explanation for lack of significant treatment effect is that the small treatment effect of losmapimod could have been masked by a much greater effect of exacerbations on vascular inflammation in COPD, particularly given this was a fibrinogen-enriched cohort, a group predicated to frequent COPD exacerbations [13]. These data therefore suggest that exacerbations are likely to increase arterial inflammation, and provides a mechanistic explanation for the increased risk of cardiovascular events in COPD patients presenting with exacerbations [25]. We did not examine correlations between systemic inflammatory biomarkers and TBR in this COPD study, since only patients with higher levels of fibrinogen were included. However, given these COPD patients with systemic inflammation do have higher levels of arterial inflammation, than what we observed in stable atherosclerosis patients, supports the concept that arterial inflammation is a plausible mechanism accounting for increased cardiovascular risk observed in chronic inflammatory conditions [23].

There was no effect of losmapimod on the co-primary endpoint of endothelial function. However, significantly greater vasodilatation following GTN administration was observed in the losmapimod group. These data suggest that losmapimod may improve endothelium-independent vasodilatation in COPD, and interestingly this effect of losmapimod has been observed previously in hypercholesterolemic patients [6]. In the hypercholesterolemia study, however, losmapimod improved endothelium-dependent *and* endothelium-independent vasodilatation, but endothelial function was assessed by forearm venous occlusion plethysmography and intra-arterial acetylcholine infusion, and the study period was considerably shorter at 28 days.

Treatment with losmapimod did not alter arterial stiffness in this study. However, this may be expected since arterial stiffness may not be related to systemic inflammation in COPD patients, at least in cross-sectional analyses [26]. Mechanical changes to the arterial wall, particularly of the large central arteries, such as calcification [27], and, or reduced elastin in the arterial wall, potentially due to the detrimental systemic effects of smoking per-se may be a more robust explanation accounting for the increased arterial stiffness reported in COPD patients.

Pharmacological inhibition of the p38 MAPK pathway is associated with dampening of circulating systemic inflammatory biomarkers particularly in the short-term, and fibrinogen has most widely been assessed in COPD, and hsCRP in cardiovascular disease. The greatest reduction in these biomarkers is generally observed in the first 2–4 weeks following MAPK inhibition, with a gradual drift up from this nadir thereafter, and often a rebound of higher levels than baseline upon cessation of treatment [3] [5]. The pattern of attenuation in our fibrinogen data is similar, with a statistically significant reduction in fibrinogen at 2–4 weeks only. In contrast, two previous studies of losmapimod in COPD, demonstrated sustained lowering of fibrinogen with losmapimod treatment, at 12 weeks [4] [28]. The trajectory of fluctuations in hsCRP across the treatment period in our small trial, which are likely due to exacerbations, highlight the difficultly of interpreting inflammatory biomarker data in this current study.

Limitations of this exploratory study are the relatively small study size which may have restricted our ability to detect a smaller treatment effect than we predicted, based on data from a different population [5]. Further, the exacerbations which occurred in this cohort, which is consistent with the current understanding that fibrinogen predicts these events, makes discriminating small anti-inflammatory effects more difficult. Moreover, we chose a 4 month treatment period, whereas losmapimod reduced arterial inflammation in atherosclerosis patients at 3 months [5], and improved endothelial function in hypercholesterolemic subjects following 1 month of treatment [6]. These data suggest that the ideal treatment duration of losmapimod may be in the acute setting, or for shorter periods of therapy, similar to the use of corticosteroids in exacerbations.

To the best of our knowledge, this is the first trial to use fibrinogen as a stratification biomarker for entry to a COPD study. The clinical implications of fibrinogen reduction previously reported in losmapimod studies in COPD are currently unclear, but fibrinogen in COPD, is associated with poor outcomes and is a more stable biomarker than CRP [29], which supported its use as a stratification biomarker in this study. Indeed, the FDA has qualified fibrinogen as a drug development tool to be used in studies where patients are at high risk of exacerbations and mortality [13], and the stable levels of fibrinogen in the EVOLUTION placebo group over 4 months, and incidence of COPD exacerbations in the study, further support its utility as a drug development tool.

In summary, 16 weeks treatment with losmapimod was well tolerated but demonstrated no effect on index vessel inflammation and only a short-term reduction in systemic inflammation biomarkers in this cohort of COPD patients with evidence of systemic inflammation. Although endothelial-independent vasodilatation responses improved with losmapimod, there was no change in endothelial-dependent vasodilatation. These findings suggest that losmapimod is unlikely to be an effective long-term treatment for the adverse cardiovascular extra-pulmonary manifestations of COPD.

## Supporting information

S1 Protocol EVOLUTION trial protocol. (PDF)

S1 Appendix CONSORT checklist EVOLUTION trial. (DOC)

S2 Appendix Raw data. (ZIP)

S1 Methods (DOCX)

S1 Table Inclusion criteria of the trial. (DOCX)

S2 Table Exclusion criteria of the trial. (DOCX)

S3 Table Breakdown of trial screen failures. (DOCX)

S4 Table Covariate data used to evaluate trial endpoints. (DOCX)

S5 Table Summary of adverse events in the placebo and losmapimod groups. (DOCX)

S1 Fig Flow-chart of trial visits and assessment. (TIFF)
