# Supplementary material for: The p38 mitogen activated protein kinase inhibitor losmapimod in chronic obstructive pulmonary disease patients with systemic inflammation, stratified by fibrinogen: A randomised double-blind placebo-controlled trial
Source: PLoS One. 2018 Mar 22;13(3):e0194197. doi: 10.1371/journal.pone.0194197 (PMC5863984; doi:10.1371/journal.pone.0194197)
Supplement: S1 Protocol — (PDF) [file pone.0194197.s001.pdf]

EVOLUTION

Clinical Trial Protocol

An **Ev**aluation **o**f **L**osmapimod in patients with Chronic Obstructive P**u**lmonary Disease (COPD) with sys**t**emic inflammat**ion** stratified using fibrinogen (EVOLUTION)

Protocol Number: EVOLUTION

Protocol Short title: Losmapimod in COPD patients stratified by fibrinogen (EVOLUTION)

REC Number: 12/EE/0135

EudraCT Number: 2011-004936-75

ISRCTN Number: NCT01541852

GSK Reference: CRT116192

Investigational Product: Losmapimod

Protocol Version: 3.1, 01 Jun 2014

Chief Investigator:	Dr Ian B. Wilkinson<br>Clinical Pharmacology Unit, Box 110<br>Addenbrookes Hospital<br>Hills Road<br>Cambridge<br>CB2 0QQ
Telephone:	01223 336898
Trial Sponsor:	Cambridge University Hospitals NHS Foundation Trust & the University of Cambridge
SAE Reporting:	01223 256623 (Fax)
Email	cctu@addenbrookes.nhs.uk
GSK SAE Reporting:	0208 754 7822 (Fax)
Email:	oax37649@gsk.com
Medical Monitor:	Professor John Cockcroft<br>Department of Cardiology,<br>Wales Heart Research Institute,<br>University of Wales College of Medicine,<br>Heath Park,<br>Cardiff, CF14 4XN<br>United Kingdom
Telephone:	029 2074 3489
Email:	cockcroftJR@cardiff.ac.uk

1 Protocol Signatures:

I give my approval for the attached protocol entitled: "An **Ev**aluation **o**f **L**osmapimod in patients with Chronic Obstructive P**u**lmonary Disease (COPD) with sys**t**emic inflammat**ion** stratified using fibrinogen (EVOLUTION)" dated 01 Mar 2013 Version 3.0

Chief Investigator

Name: Dr Ian B. Wilkinson

Signature: ____

Date: ________________________________

Site Signatures

I have read the attached protocol entitled "An **Ev**aluation **o**f **L**osmapimod in patients with Chronic Obstructive P**u**lmonary Disease (COPD) with sys**t**emic inflammat**ion** stratified using fibrinogen (EVOLUTION)" dated 01 Mar 2013 Version 3.0 and agree to abide by all provisions set forth therein.

I agree to comply with the International Conference on Harmonisation Tripartite Guideline on Good Clinical Practice; The European Clinical Trials Directives 2001/20/EC and 2005/28/EC.

I agree to ensure that the confidential information contained in this document will not be used for any other purpose other than the evaluation or conduct of the clinical investigation without the prior written consent of the Sponsor

Principal Investigator

Name: Dr Joseph Cheriyan

Signature: ____

Date: ________________________________

2 Trial Steering Committee and Protocol Contributors

Investigators:	
**Dr Ian B. Wilkinson**	Clinical Pharmacology Unit, University of Cambridge
**Dr Joseph Cheriyan**	Clinical Pharmacology Unit, Cambridge University Hospitals NHS Foundation Trust
**Dr Carmel M. McEniery**	Clinical Pharmacology Unit, Cambridge University Hospitals NHS Foundation Trust
**Dr Jonathan S. Fuld**	Department of Respiratory Medicine, Cambridge University Hospitals NHS Foundation Trust
**Dr James H.F. Rudd**	Department of Cardiology, University of Cambridge
**Dr Martin Graves**	Department of Radiology, University of Cambridge
**Ruth Tal-Singer**	Galaxy Initiative, Respiratory Therapy Unit, GlaxoSmithKline
**Professor Mike Polkey**	Dept of Respiratory Medicine, Royal Brompton NHS Foundation Trust
**Professor David A Lomas**	Department of Respiratory Medicine, University of Cambridge
**Dr Philip S Murphy**	Senior Director, Clinical Imaging at GlaxoSmithKline
**Professor Peter Calverley**	Independent Chair, Trial Steering Committee, Department of Respiratory Medicine, University Hospital Aintree
**Professor John Cockcroft**	Medical Monitor, Department of Cardiology, Wales Heart Research Institute, University of Wales College of Medicine

**Trial Statistician:**
Dr Simon Bond<br>Cambridge Clinical Trials Unit (Box 111)<br>Addenbrookes Hospital, Hills Road<br>Cambridge, CB2 0QQ<br>Telephone: 01223 596475 Fax: 01223 596471<br>e-mail: simon.bond@addenbrookes.nhs.uk
**Trial Coordination:**
Dr Katan Patel<br>Cambridge Clinical Trials Unit (Box 111)<br>Addenbrookes Hospital, Hills Road<br>Cambridge, CB2 0QQ<br>Telephone: 01223 349243 Fax: 01223 596471<br>e-mail: katan.patel@addenbrookes.nhs.uk
**Sponsor:**
Cambridge University Hospitals NHS Foundation Trust and University of Cambridge<br>Dr John Bradley / Helen Atkinson<br>Director of Research & Development / Assistant Director<br>Research & Development Department (Box 277)<br>Addenbrooke's Hospital, Hills Road<br>Cambridge, CB2 0QQ<br>Telephone: 01223 245151<br>e-mail: cctu@addenbrookes.nhs.uk

Amendment History

Version No.	History	Date
V1.0	Final Protocol	01 Jan 2012
V1.1	Minor Amendment	16 Mar 2012
V1.2	Minor Amendment	26 Apr 2012
V2.0	Substantial Amendment	01 Nov 2012
V3.0	Substantial Amendment	01 Mar 2013
V**3.1**	Minor Amendment	01 Jun 2014

3 Table of Contents

1 Protocol Signatures: ... 2
2 Trial Steering Committee and Protocol Contributors ... 3
3 Table of Contents ... 5
4 Abbreviations ... 7
5 Trial Synopsis ... 9
6 Trial Flow Chart ... 15
7 Introduction ... 16
7.1 Background ... 16
8 Rationale for Trial ... 16
9 Trial Design ... 18
9.1 Statement of design ... 18
9.2 Number of Sites ... 18
9.3 Number of Participants ... 19
9.4 Trial duration ... 19
9.5 Trial objectives ... 19
9.6 Trial endpoints ... 19
10 Selection and withdrawal of participants ... 20
10.1 Inclusion Criteria ... 20
10.2 Exclusion Criteria ... 20
10.3 Method of Blinding ... 21
10.4 Emergency Unblinding ... 22
10.5 Participant Withdrawal Criteria and Stopping Criteria ... 22
11 Trial Treatments ... 23
11.1 Data from Non-Clinical Studies ... 23
11.2 Clinical Data ... 23
11.3 Dose Rationale ... 26
11.4 IMP Dosage and Administration ... 26
11.5 Descriptions: Losmapimod and matching Placebo ... 27
11.6 Losmapimod drug interactions ... 28
11.7 Handling and Storage of IMP ... 28
11.8 IMP Accountability ... 28
11.9 Treatment Assignment ... 28
11.10 Treatment Compliance ... 29
11.11 Concomitant Medications and Non-Drug Therapies ... 29
11.12 Treatment after EVOLUTION Trial ... 29
11.13 Treatment of IMP Overdose ... 29
11.14 Legal status of the drug ... 29
12 Procedures and Trial Assessments ... 29
12.1 Trial Visits ... 29
12.2 Clinical laboratory assessments ... 32
12.3 Pregnancy ... 33
12.4 Imaging ... 33
12.5 Non Imaging End Points ... 34
12.6 Six minute walk test ... 35
12.7 Blood Sampling ... 35
12.8 Trial restrictions ... 36
12.9 Safety ... 36
13 Assessment of Safety ... 37
13.1 Safety Reporting Definitions ... 37
13.2 Evaluating AEs and SAEs ... 39
13.3 Expected Serious Adverse Events ... 40
13.4 Recording and evaluation of AEs and SAEs ... 40
13.5 Reporting of Suspected Unexpected Serious Adverse Reactions (SUSARs)41

14 Toxicity – Emergency Procedures 42
15 Evaluation of Results 45
16 Statistics 46
16.1 Statistical methods 46
16.2 Analysis Populations 47
16.3 Derived and Transformed Data 48
16.4 Number of Participants to be enrolled 48
16.5 Criteria for the termination of the trial 48
16.6 Procedure to account for missing or spurious data 48
16.7 Definition of the end of the trial 49
17 Data handling and record keeping 49
17.1 eCRF 49
17.2 Source Data 49
17.3 Data Protection 49
18 Trial Steering and Data Monitoring Committee 49
19 Ethical & Regulatory considerations 50
19.1 Consent 50
19.2 Ethical committee review 50
19.3 Regulatory Compliance 50
19.4 Protocol Amendments 50
19.5 Declaration of Helsinki and ICH Good Clinical Practice 51
19.6 GCP Training 51
20 Sponsorship, Financial and Insurance 51
21 Monitoring, Audit & Inspection 51
22 Protocol Compliance and Breaches of GCP 51
23 Publications policy 52
24 References 53
25 Appendices 55
25.1 Appendix 1 - Trial Management / Responsibilities 55
25.1 Time and Events Table (Table 7) 56

4 Abbreviations

6MWT	6 Minute Walk Test
AE	Averse Event
Aix	Augmentation Index
ALT	Alanine aminotransferase
ATP	Adenosine Triphosphate
AST	Aspartate aminotransferase
ATS	American Thoracic Society
AUC	Area Under the plasma drug Concentration versus time curve; a measure of drug exposure
AR	Adverse Reaction
ATP	Potent Adenosine Triphosphate
BCRP	Breast Cancer Resistance Protein
BD	Twice Daily
BMI	Body Mass Index
BP	Blood Pressure
CABG	Coronary Artery Bypass Graft
CCTU	Cambridge Clinical Trials Unit
CI	Chief Investigator
C_{MAX}	The peak serum concentration of a therapeutic drug
COPD	Chronic Obstructive Pulmonary Disease
CPK	Serum Creatine Phosphokinase
CRF	Case Report Form
CTA	Clinical Trial Application or Authorisation
CTIMP	Clinical Trial of an Investigational Medicinal Product
DoB	Date of Birth
DSUR	Development Safety Update Report
DCCT	Diabetes Control and Complications Trial
E_{max}	Maximum effect
ECG	Electrocardiogram
eCRF	Electronic Case Report Form
EMA	European Medicines Agency
ERICA	Evaluation of the Role of Inflammation in Chronic Airways disease (COPD)
FDA	Food and Drug Administration
FDG	Fluorodeoxyglucose
FEV1	Forced Expiratory Volume in one second
FVC	Forced Vital Capacity
FFMI	Free Fat Mass Index
FMD	Flow Mediated Dilation
GP	General Practitioner
GSK	GlaxoSmithKline
GTN	glyceryl trinitrate
HDL	High Density Lipoprotein
HDPE	High-Density Polyethylene
HIV	Human Immunodeficiency Virus
HPLC	High Performance Liquid Chromatography
HR	Heart Rate
IB	Investigator Brochure
ICF	Informed Consent Form
IFCC	International Federation of Clinical Chemistry and Laboratory Medicine
IMP	Investigational Medicinal Product

IMPD	Investigational Medicinal Product Dossier
ISF	Investigator Site File
IUD	Intrauterine device
IV	Intravenous
LDL	Low Density Lipoproteins
LFT	Liver Function Tests
LPS	LipoPolySaccharide
msec	Milliseconds
MAP	Mean Arterial Pressure
MAPK	Mitogen-Activated Protein Kinase
MHRA	Medicines and Healthcare products Regulatory Agency
MI	Myocardial Infarction
MRI	Magnetic Resonance Imaging
NF	National Formulary
NHS	National Health Service
NOAEL	No observed adverse effect level
PCI	Percutaneous Coronary Intervention
PET/CT	Positron Emission Tomography – Computed Tomography
PI	Principle Investigator
PWV	Pulse Wave Velocity
QTc	Corrected QT interval of electrocardiogram
RBH	Royal Brompton & Harefield Foundation NHS Trust
REC	Research Ethics Committee
SAE	Serious Adverse Event
SAR	Serious Adverse Reaction
SD	Standard Deviation
SmPC	Summary of Product Characteristics
SNIP	Sniff Nasal Inspiratory Pressure
SUSAR	Suspected Unexpected Serious Adverse Reaction
SUV	Standard Uptake Value
TBR	Tissue to Background Ratio
TIA	Transient Ischaemic Attack
Tmax	Time to maximum plasma concentration
TNFα	Tumour Necrosing Factor alpha
TSC	Trial Steering Committee
TMF	Trial Master File
TPM	Trial Procedure Manual
TSC	Trial Steering Committee
ULN	Upper limit of normal
USP	United States Pharmacopeia
WBC	White Blood Cells

5 Trial Synopsis

Full Title of Clinical Trial	An **Ev**aluation **o**f **L**osmapimod in patients with Chronic Obstructive P**u**lmonary Disease (COPD) with sys**t**emic inflammat**ion** stratified using fibrinogen (EVOLUTION)
Short Title of Clinical Trial	Losmapimod in COPD patients stratified by fibrinogen (EVOLUTION)
Sponsor Name	Cambridge University Hospitals NHS Foundation Trust and University of Cambridge
EudraCT number for proposed trial	2011-004936-75
Medical condition or disease under investigation	COPD (Chronic Obstructive Pulmonary Disease)
Purpose of clinical trial	To test the hypothesis that stratifying a COPD population on plasma fibrinogen improves the ability to observe a response to an anti-inflammatory treatment in the vasculature and lungs. In this trial, Losmapimod will be used as a tool to test the hypothesis of stratification.
Primary objectives	To determine the effect of Losmapimod in COPD patients on vascular structure and function as assessed by<br>1. Vascular inflammation<br>2. Endothelial function<br>3. Arterial structure and plaque characteristics
Secondary objective (s)	To determine the effects of Losmapimod on<br>1. Inflammation in lung tissue<br>2. Inflammation in fat<br>3. Arterial stiffness<br>4. Blood biomarkers of inflammation<br>5. Indices of lung function and physical performance<br>6. Body composition and fat<br>7. Safety and tolerability
Trial Design	This is a randomised, repeat-dose, double-blind, placebo-controlled, parallel-group trial

Trial Endpoints	**Primary:**<br>Change in vascular inflammation (using derivatives of Standard Uptake Value (SUV) and Tissue to Background Ratio (TBR)) in the carotid and aortic arteries of participants following 16 weeks of treatment.<br>Change in flow mediated dilatation post-ischaemia and/or flow mediated dilatation post-glyceryl trinitrate, following 16 weeks of treatment.<br>Change in atheromatous plaque and vessel wall characterisation using MRI and/or FDG-PET/CT, following 16 weeks of treatment (MRI is an optional sub-study and analysis will be based on a sufficient number of datasets).<br>**Secondary:**<br>Change in lung inflammation assessed by FDG-PET/CT, following 16 weeks of treatment.<br>Change in visceral and subcutaneous fat inflammation and serum/plasma measures of systemic inflammation, following 16 weeks of treatment.<br>Change in arterial stiffness measures (aortic pulse wave velocity and augmentation index), following 16 weeks of treatment.<br>Change in baseline-corrected blood concentration of biomarkers.<br>Change in spirometry (FEV_1, FVC, FEV_1/FVC ratio) and maximal SNIP and 6MWT following 16 weeks of treatment.<br>Change in body composition as measured by BMI and FFMI, following a 16 week treatment.<br>Safety and tolerability parameters, including physical examination, blood pressure, heart rate, 12-lead electrocardiogram (ECGs) recordings, clinical laboratory tests, lung function, and adverse event reporting.
Sample Size	A sufficient number of participants will be recruited to complete approximately 60 participant data sets suitable for the primary statistical analysis (approximately 30 per arm).
Summary of inclusion criteria	• Male or female patients between 50 and 85 years of age inclusive at screening, with a body weight ≥ 45 kg and BMI ≤ 35 kg/m^2.<br>• Patients with a clinical diagnosis of COPD GOLD Stages 1, 2, 3 or 4 & U<br>• FEV_1/FVC < 0.7 post-bronchodilator

	• Patient is a current smoker or an ex-smoker with a smoking history of at least 10 pack years.<br>• Baseline fibrinogen value of >2.8 g/L (Klauss method).<br>• ALT < 2xULN at screening; alkaline phosphatase and bilirubin ≤ 1.5xULN.<br>• Patients must have a QTc <450 msec on screening (V1) ECG (using average value of triplicate ECGs). For patients with complete Right bundle branch block, the QTc must be <480msec on Screening V1 ECG. Patients with other ECG findings will be excluded if warranted at the discretion of the CI/PI. QTc readings will be QTcF.<br>• (For the optional MRI sub-study (n/a if patient does not consent to MRI)): Patients who fulfil local imaging centre requirements will be enrolled.
Summary of exclusion criteria	• Inability in the opinion of the PI to provide Informed Consent.<br>• A cardiovascular event in the last 6 months (i.e. acute coronary syndrome, unstable angina, CABG, PCI, stroke, MI, carotid endarterectomy).<br>• Patients on daunorubicin, doxorubicin, topotecan, mitoxantrone.<br>• Previous lung reduction surgery.<br>• Patients with known clinically significant pulmonary diagnoses in which inflammation is thought to play a role including diagnosis of bronchiectasis, sarcoidosis, lung fibrosis, interstitial lung disease, or α1-antitrypsin deficiency.<br>• A positive pre-trial Hepatitis B surface antigen or positive Hepatitis C antibody result within 3 months of screening.<br>• Current or chronic history of liver disease, or known hepatic or biliary abnormalities (with the exception of Gilbert's syndrome or asymptomatic gallstones).<br>• Patients with known chronic infections such as HIV or known active tuberculosis.<br>• Patients with rheumatoid arthritis, connective tissue disorders and other conditions known to be associated with active chronic inflammation (e.g. Inflammatory Bowel Disease).<br>• Insulin controlled Type 1 or Type 2 diabetics.

	• Diabetics on oral hypoglycaemics/diet with HbA1c (DCCT) > 8% (OR HbA1c (IFCC) > 64 mmol/mol), at screening. [note: fasting glucose will be checked again at first FDG-PET/CT scan, and if glucose > 11mmol/L at that visit, patients will be excluded from trial]<br>• Participation in a previous research trial in the last 3 years which involved exposure to significant ionising radiation (i.e. cumulative research radiation dose >5 mSv)<br>• History of malignancy within the past 5 years (with the exception of localized carcinoma of the skin that has been resected for cure).<br>• Previous exposure to Losmapimod.<br>• Patients who have donated more than 500 mL of blood within 2 months prior to the trial medication administration, Visit 3 (Day 1).<br>• Participation in a clinical trial where the patient has received a drug or new chemical entity within 30 days or 5 half-lives, or twice the duration of the biological effect of the drug (whichever is longer) prior to the first dose of trial medication, Visit 3 (Day1).<br>• History of alcohol/drug abuse or dependence within 6 months of screening, Visit 1 (Days -45 to -14).<br>• Women of childbearing potential are excluded from this trial. Women must be of non-childbearing potential [i.e. either postmenopausal or documented hysterectomy and/or bilateral oophorectomy – tubal ligation is not sufficient]. For the purposes of this trial, postmenopausal is defined as being amenorrhoeic for greater than 2 years with an appropriate clinical profile, e.g. age appropriate, history of vasomotor symptoms.<br>• An unwillingness of male patients to abstain from sexual intercourse with pregnant or lactating women; or an unwillingness of the participant to use a condom/spermicide in addition to having their female partner use another form of contraception such as an intrauterine device (IUD), diaphragm with spermicide, injectable progesterone, sub-dermal implants or a tubal ligation.<br>• Any medical history or clinically relevant abnormality that is deemed by the principal investigator and/or medical monitor to make the patient ineligible for inclusion because of a safety concern.

	• Use of systemic corticosteroids (oral or IV) 4 weeks prior to visit 2 (Day -14 to -1).
Investigational Medicinal Product and dosage	Losmapimod 7.5mg BD (GW856553)
Comparator product(s)	Placebo
Route(s) of administration	Oral
Maximum duration of treatment of a Patient	112 days
Procedures: Screening & enrolment	• Participants will attend the unit for a screening visit within 45 days prior to administration of the first dose of trial medication. Written informed consent must be obtained prior to performance of any protocol specific procedures. The following procedures will be performed:<br>• Medical history including smoking history<br>• Current medication history prior to the screening visit.<br>• Demography (date of birth, age, gender and race)<br>• Physical examination including height, weight, BMI, seated blood pressure and heart rate<br>• Spirometry post bronchodilator<br>• Standard, supine 12-lead ECG (triplicate)<br>• Following an overnight fast, blood (approximately 55ml) and urine will be collected for clinical chemistry, haematology, HIV, Hepatitis B surface antigen, Hepatitis C antibody, urinalysis.
Trial period:	Participants will be entered into the trial for a period of up to 171 Days (approximately 6 Months).<br><br>Participants will be asked to attend for up to 8 visits. These will consist of 2 scanning visits at V2 & V8. V3, V4, V5, V6, V7 and V9 participants will have a number of tests performed as listed below, they will also receive study medication during this period.
End of Trial	The end of trial definition will be 3 months after last participant last visit.<br>• Physical examination including seated blood pressure (BP) and heart rate (HR).<br>• Blood (approximately 55mL) and urine will be collected for clinical chemistry, haematology, urinalysis.<br>• Bloods for biomarkers.<br>• Adverse event and concomitant medication check.
Procedures for safety monitoring during trial	Clinical safety data is a secondary endpoint of this trial. The safety and tolerability of protocol-specified treatments will be assessed by physical exam findings, 12-lead ECGs recordings, vital

	signs (blood pressure and heart rate) measurements, clinical laboratory tests (including LFT), clinical monitoring /observation and spontaneous and elicited adverse event reporting. Methodology for safety assessments will be on file at the clinical research unit.<br><br>The Trial Steering Committee (TSC) will provide overall supervision for the trial, to ensure that it is conducted in accordance with the protocol and GCP and to provide advice through its independent chairman.
Criteria for withdrawal of patients on safety grounds	Participants will be withdrawn from the trial if they experience a SUSAR. At the discretion of the PI/CI, participants will be withdrawn if they experience a SAE, which would affect their ability to participate in the trial.

6 Trial Flow Chart

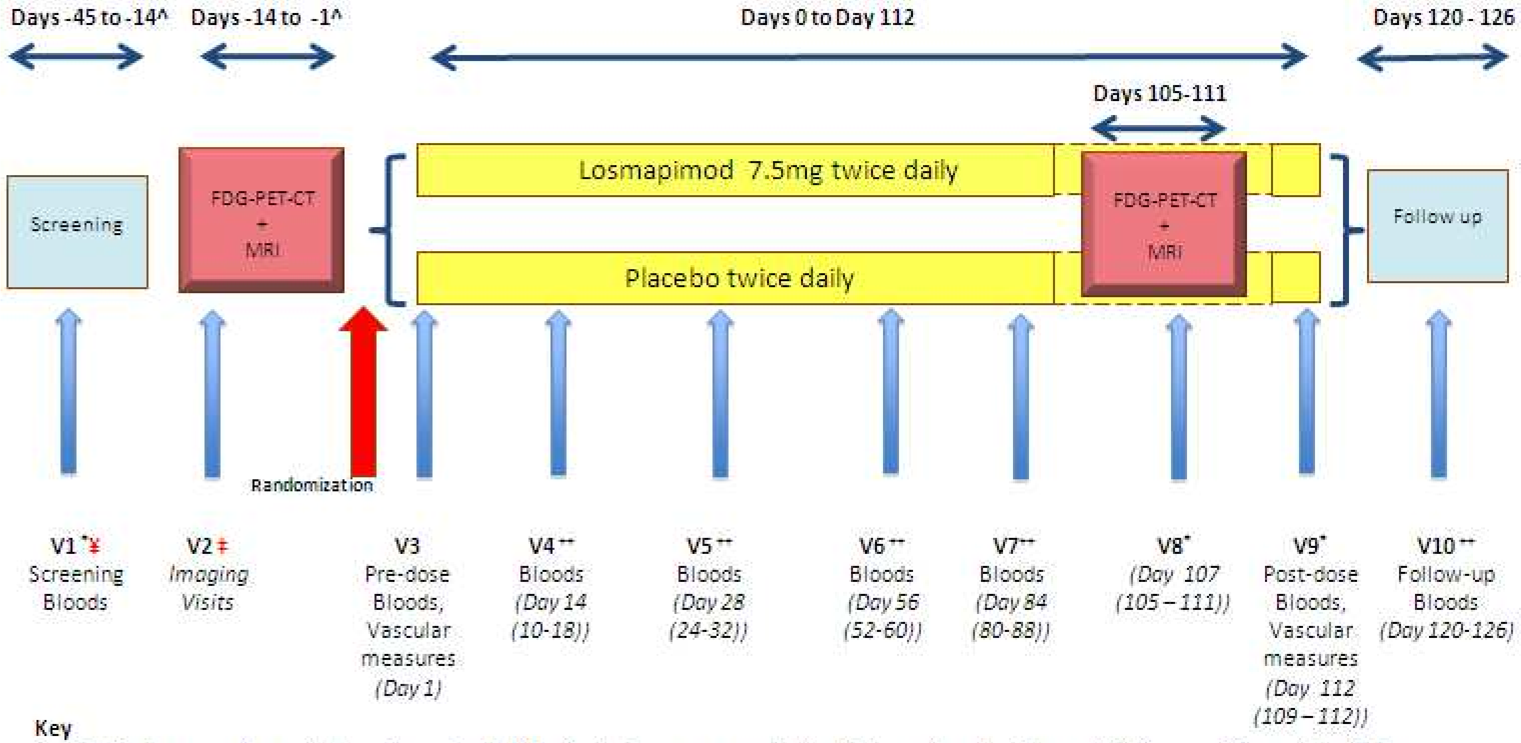

Key

^ The 45 day screening period may be extended if patients have an exacerbation that requires steroid or antibiotic use. This period will be extended by at least the equivalent time of steroid use added onto the 45 day period or up to the discretion of the PI where clinical judgement is required.

¥ Patients who have failed screening because of changes to trial inclusion/exclusion criteria or did not attend screening due to medical reasons (i.e. an exacerbation) and their medical status has now changed may be rescreened if they are keen to participate in the trial and happy to undergo a repeat screening, at the discretion of the PI and based on clinical judgement

‡ Patients who have attended the imaging visit but do not progress within 14 days onto the trial due to the necessity to investigate abnormalities on the PET-CT / MRI scan may be considered for subsequent entry onto the trial without undergoing a repeated imaging visit, if performed within a reasonable timeframe, and at the discretion of the PI and based on clinical judgement.

* Fixed between the dates specified (unless file noted for a specific reason) ** ± 96 Hours or as indicated

All the above mentioned procedures as per key can take place ± 96h or as indicated. V8, the FDG-PET/CT and MRI imaging visit can occur at any time between Days 105-111. V9 can occur any time from Day 109-112. However, if there is a specific reason why this may not occur, the deviation will be logged in the Trial File. In some cases, the FDG-PET/CT and MRI scans (Imaging Visits 2 and 8) will occur on the same day but it is very likely that scans be done on separate visits during the intervals indicated above. In the event LFT abnormalities are seen, LFT monitoring may need to occur more frequently, as determined by the investigator and medical monitor.

7 Introduction

7.1 Background

Chronic obstructive pulmonary disease (COPD) is the fourth leading cause of death globally and the only major cause that is predicted to increase in the coming decades[1,2]. Although COPD is initially a pulmonary disease (traditionally caused by cigarette smoke, but increasingly by biomass fuel exposure), it is a complex condition in which outcome is in large part, driven by cardiovascular and systemic components. Consistent with this, although current bronchodilator-based interventions have demonstrated reduced exacerbation rates, this has only addressed part of the burden of disease. In particular, drugs currently used neither reduce the rate of disease progression nor mortality, creating a space in the market for products which could do so. This unmet need and the implied requirement for new strategies for the development of non-bronchodilator therapies for COPD have been recognized by Regulatory agencies as evident from recent guidance documents from the EMA[3] and FDA[4].

The role of inflammation in COPD is well-recognised. A range of inflammatory markers and cytokines are raised in COPD patients, and relate to the severity of lung function and important clinical outcomes such as admission, cardiovascular events and death. Elevated fibrinogen (indicating a systemic 'inflammatory' phenotype) is a robust, stable biomarker that occurs in at least 25% of patients with COPD, and is associated with increased morbidity and mortality. Inflammation modulates large artery stiffness, an independent determinant of cardiovascular mortality, and anti-inflammatory therapies reduce arterial stiffness[11], providing a novel mechanism by which inflammation may drive cardiovascular risk.

Losmapimod is a ATP binding site inhibitor of p38α MAPK and is currently being developed by GSK for a variety of inflammatory conditions, including COPD.

The ERICA programme is a series of studies designed to clarify the role of inflammation in extra-pulmonary disease manifestations in COPD. This trial will assess the role of stratifying patients on the basis of systemic inflammatory biomarkers against parameters of vascular, pulmonary and skeletal muscle phenotypes. This may enable the development of better biomarkers to design smaller, more efficient phase I-III clinical trials of novel drugs that target inflammatory COPD subsets thereby reducing attrition of new medicines.

8 Rationale for Trial

Data from a GlaxoSmithKline (GSK) sponsored observational cohort trial, ECLIPSE, confirmed that cardiovascular disease is common in COPD[5]. Pooled data from large clinical trials of drugs currently in use suggest an equivalent probability of participants dying from cardiovascular or respiratory causes[6]. Moreover, large cohort studies suggest that drugs active against cardiovascular manifestations (for example statins or ACE inhibitors) both reduce mortality and admission with acute exacerbation of COPD[7]. The precise underlying mechanisms by which this may occur have not been completely elucidated, and these may be exaggerated in certain COPD sub-types.

The cardiovascular manifestations in COPD may vary in their pathophysiological origins. Reduced nitric oxide bioavailability (endothelial dysfunction), aortic stiffening and direct vascular inflammation are all potential candidates, as is accelerated atheroma formation. Elevated fibrinogen (indicating a systemic 'inflammatory' phenotype) is a robust, stable biomarker that occurs in at least 25% of patients. It is associated with increased mortality

and morbidity (unpublished data from ECLIPSE). Indeed, this subset has an almost three-fold elevated risk of cardiovascular admissions[8] suggesting that systemic inflammation may accelerate cardiovascular disease[9].

Atherosclerosis is characterized by the accumulation of lipids, inflammatory cells and connective tissue cells within the arterial wall. It is a chronic progressive disease with a long asymptomatic phase. Over the past decade, the accumulated evidence indicates that inflammation plays a central role in the pathophysiology of atherosclerosis. Indeed, the p38 MAPK cascade appears to play a key role in the initiation and progression of inflammatory diseases. It is therefore likely that inhibition of this enzyme, and its ensuing signal transduction pathway, could attenuate the initiation and maintenance of atherosclerotic events, suggesting a possible therapeutic potential for a p38 MAPK inhibitor in modulating vascular inflammation associated with COPD.

Rupture of atherosclerotic plaques can lead to myocardial infarctions and cause sudden cardiac deaths. Such vulnerable plaques have typical histopathological features that include the presence of a thin fibrous cap, extensive lipid core and an associated inflammatory infiltrate[10,11]. Macrophages, key inflammatory cells, abound in these plaques and imaging of these macrophages is an appealing approach to detect plaque inflammation that may lead to clinical complications. Recently, FDG-PET/CT has demonstrated success in detecting plaque inflammation within the aorta, carotid and vertebral arteries and has been validated histologically, as correlating with macrophage content and/or activity, both in human and animal models of disease[11,12]. COPD patients have previously been demonstrated to have increased FDG uptake suggesting increased background vascular inflammation in this group[13].

The main goals of this experimental trial are to test the hypothesis that stratifying a COPD population on plasma fibrinogen improves the ability to observe response to an anti-inflammatory treatment known to reduce fibrinogen as well as reduce inflammatory activity in the extra-pulmonary and pulmonary spaces in these patients. Therefore, Losmapimod will be used as a tool to test the hypothesis that it improves vascular health in inflamed COPD patients.

FDG-PET/CT imaging will be used to assess Losmapimod's effects on inflammatory activity within aortic and carotid vessels, as well as pulmonary and other extra-pulmonary spaces in an enriched population of COPD patients, characterised by high plasma fibrinogen at baseline. A panel of biomarkers, including endothelial function will also be measured. The American Heart Association's conventional classifications of plaques have been amended to allow MRI classification of plaque. In addition, T2 mapping is a relatively new technique that has been developed to detect and quantify oedema and hydration status of the aortic wall. We aim to assess the effect of p38 inhibition on plaque and aortic wall characteristics in an MRI (T1 and T2 weighted) sub-trial of the carotids and aorta. Endothelial function will be assessed using flow mediated dilatation. Arterial stiffness (as measured by aortic pulse wave velocity) has been shown to be a powerful predictor of cardiovascular mortality. Therefore this trial will provide an opportunity to measure aortic pulse wave velocity to determine the extent to which a p38 MAP kinase inhibitor can mediate changes after a 16 week treatment period in COPD patients.

Inflammation mediates various stages of atherosclerosis development and progression from initial leukocyte recruitment to eventual rupture of the high risk plaque. ^{18}F-deoxyglucose (^{18}FDG) is a synthetic tracer that mimics the biochemical behavior of the natural glucose molecule. ^{18}FDG positron emission tomography (^{18}FDG-PET) has been established to be useful for detecting not only tumor cells, but also inflammatory cells that are metabolically active.

Arterial FDG uptake was first noted in patients undergoing PET for cancer staging[14]. After balloon aortic injury in a rabbit model of atherosclerosis, FDG uptake in atherosclerotic regions correlated strongly with macrophage content (r=0.93, p<0.002)[15] findings confirmed by others. In the first prospective trial in man, levels of inflammation measured as accumulation rate of FDG were 27% greater in the culprit carotid after recent stroke or TIA than in the contra lateral vessel[16] though with contemporary therapy this difference is not apparent beyond 38 days of the event[17]. Autoradiography of carotid plaque specimens reported in the same paper confirmed co-localisation of tracer to regions of macrophage staining. Further work has confirmed FDG uptake correlates with areas of macrophage (CD68+) staining (r=0.96, p<0.001), and is independent of plaque thickness, area or luminal stenosis[18, 19]. FDG uptake is thought to reflect activation status of macrophages[21,22] though recent work by Folco[23] suggests that hypoxia known to exist within atherosclerotic plaque[24] may also be an important determinant of glucose (and hence FDG) uptake into these cells. Nevertheless, FDG-PET remains the best validated tracer in molecular atherosclerosis imaging.

FDG accumulation within the arterial wall could be used to quantify the therapeutic effectiveness of Losmapimod. As a functional imaging modality, FDG positron emission tomography (PET) alone may have some limitations in quantifying the extent of atherosclerosis, as it does not give any information about the morphology of the arterial wall, such as thickness or volume and location and structural details of plaque(s). Therefore a combined PET/CT scanner will be used for this project. Combined PET/CT scanners have several advantages over standalone machines. Firstly, they allow accurate (within 1mm) co-registration of the PET images, with no need for further co-registration software. Secondly, accurate attenuation correction of the PET dataset is possible using the CT images. This shortens the scan time for the patient compared to stand alone PET and CT scanners.

Thirdly, the use of PET/CT combined scanners has been evaluated in a similar patient group and has been found to be a reliable and reproducible technique[14]. In addition, due to limits on radiation exposure in research Participants, PET-CT will not allow for the accurate characterisation and delineation of plaque from vessel wall. As MRI works on the principle of differentiating water and fat resonance, it is the best imaging modality for identifying anatomical plaques without increasing radiation. Scans will be performed on GE scanners in Cambridge (or Siemens in London), thereby allowing co-registration of PET-CT with MRI.

9 Trial Design

9.1 Statement of design

This is a randomised, repeat-dose, double-blind, placebo-controlled, parallel-group trial.

9.2 Number of Sites

There will be 2 sites taking part namely Cambridge University Hospitals NHS Foundation Trust and Royal Brompton & Harefield Foundation NHS Trust. The co-ordination of the clinical trial will be performed by the Cambridge Clinical Trials Unit, Cambridge University Hospitals NHS Foundation Trust. The imaging centre the Royal Brompton & Harefield NHS Foundation Trust is performed by Imanova clinical Imaging Centre, Burlington Danes building Imperial College London, Hammersmith Hospital.

9.3 Number of Participants

A sufficient number of patients with COPD will be enrolled across the 2 participating sites, so that approximately 60 participants with data suitable for the primary statistical analysis (approximately 30 per arm) complete the trial.

9.4 Trial duration

The expected duration of the trial is up to 2.5 years. Participants will be enrolled into the trial over a period of approximately 24 weeks. A full break down of screening through to follow-up can be found in Section 6 page 15 -Trial Flow Chart.

9.5 Trial objectives

9.5.1 Primary Objectives

To determine the effect of Losmapimod in COPD patients on vascular structure and function as assessed by:

1. Vascular inflammation
2. Endothelial function
3. Arterial structure and plaque characteristics (optional sub-study)

9.5.2 Secondary Objectives

To determine the effects of Losmapimod on:

1. Inflammation in lung tissue
2. Inflammation in fat
3. Arterial stiffness
4. Blood biomarkers of inflammation
5. Indices of lung function and physical performance
6. Body composition and fat
7. Safety and tolerability

9.6 Trial endpoints

9.6.1 Primary endpoints

Change in vascular inflammation (using derivatives of Standard Uptake Value (SUV) and Tissue to Background Ratio (TBR) in the carotid and aortic arteries of patients following 16 weeks of treatment.

Change in flow mediated dilatation post-ischaemia and/or flow mediated dilatation post-Glyceryl trinitrate, following 16 weeks treatment.

Change in atheromatous plaque and vessel wall characterisation using MRI and/or FDG-PET/CT, following 16 weeks of treatment. MRI analysis will be dependent on a sufficient number of datasets since this was an optional sub-study (please see section 15 of the protocol for further details).

9.6.2 Secondary endpoints

Change in lung inflammation assessed by FDG-PET/CT, following 16 weeks of treatment.

Change in visceral and subcutaneous fat inflammation and serum/plasma measures of systemic inflammation, following 16 weeks of treatment.

Change in arterial stiffness measures (aortic pulse wave velocity and augmentation index), following 16 weeks of treatment.

Change in baseline corrected blood concentration of biomarkers.

Change in spirometry (FEV_1, FVC, FEV_1/FVC ratio), maximal sniff nasal inspiratory pressures and 6 MWT following 16 weeks of treatment.

Change in body composition as measured by BMI and FFMI, following 16 weeks of treatment.

Safety and tolerability parameters, including physical examination, blood pressure, heart rate, 12-lead electrocardiogram (ECGs) recordings, clinical laboratory tests, lung function, and adverse event reporting.

10 Selection and withdrawal of participants

10.1 Inclusion Criteria

A patient will be eligible for inclusion in this trial only if all of the following criteria apply:

1. Male or female patients between 50 and 85 years of age inclusive at screening, with a body weight ≥ 45 kg and BMI ≤35 kg/m^2.
2. Patients with a clinical diagnosis of COPD with GOLD Stages 1, 2, 3 or 4, or GOLD-U. Gold U are patients with a clinical history consistent with COPD and a reduced FEV1 but a preserved (i.e. >0.7) FEV1/VC ratio in the absence of an alternative diagnosis. For the purpose of EVOLUTION, establishing an absence of an alternative diagnosis will normally include as a minimum physical examination of the thoracic cage, a normal chest radiograph and the demonstration of a maximal sniff nasal inspiratory pressure >50 cm H_2O
3. Patient has FEV_1/FVC < 0.7 post-bronchodilator.
4. Patient is a smoker or an ex-smoker with a smoking history of at least 10 pack years (1 pack year = 20 cigarettes smoked per day for 1 year or equivalent).
5. Baseline fibrinogen value of >2.8 g/L (Klauss method)
6. ALT < 2xULN at screening; alkaline phosphatase and bilirubin ≤ 1.5xULN at screening (isolated bilirubin >1.5xULN is acceptable if bilirubin is fractionated and direct bilirubin <35%).
7. Patients must have a QTc <450 msec on screening (V1) ECG (using average value of triplicate ECGs). For patients with complete Right bundle branch block, the QTc must be <480msec on Screening V1 ECG. Patients with other ECG findings will be excluded if warranted at the discretion of the CI/PI. QTc readings will be QTcF.
8. (For the optional MRI sub-study (n/a if patient does not consent to MRI)): Patients who fulfil local imaging centre requirements will be enrolled.

10.2 Exclusion Criteria

The presence of any of the following will preclude patient inclusion:

1. Inability in the opinion of the PI to provide Informed Consent.
2. A cardiovascular event in the last 6 months (i.e. acute coronary syndrome, unstable angina, CABG, PCI, stroke, MI, carotid endarterectomy).
3. Patients on daunorubicin, doxorubicin, topotecan, mitoxantrone.
4. Previous lung reduction surgery.
5. Patients with known clinically significant pulmonary diagnoses in which inflammation is thought to play a role including diagnosis of bronchiectasis, sarcoidosis, lung fibrosis, interstitial lung disease, or α1-antitrypsin deficiency.
6. A positive pre-trial Hepatitis B surface antigen or positive Hepatitis C antibody result within 3 months of screening.

7. Current or chronic history of liver disease, or known hepatic or biliary abnormalities (with the exception of Gilbert's syndrome or asymptomatic gallstones).
8. Patients with known chronic infections such as HIV or known active tuberculosis.
9. Patients with rheumatoid arthritis, connective tissue disorders and other conditions known to be associated with active chronic inflammation (e.g. Inflammatory Bowel Disease).
10. Insulin controlled Type 1 or Type 2 diabetics.
11. Diabetics on oral hypoglycaemics/diet with HbA1c (DCCT) > 8% (OR HbA1c (IFCC) > 64 mmol/mol), at screening. [note: fasting glucose to be checked again at first FDG-PET/CT scan, and if glucose > 11mmol/L at that visit, patients will be excluded from trial]
12. Participation in a previous research trial in the last 3 years which involved exposure to significant ionising radiation (i.e. cumulative research radiation dose >5 mSv)
13. History of malignancy within the past 5 years (with the exception of localized carcinoma of the skin that has been resected for cure).
14. Previous exposure to Losmapimod.
15. Patients who have donated more than 500 mL of blood within 2 months prior to the trial medication administration, Visit 3 (Day 1).
16. Participation in a clinical trial where the patient has received a drug or new chemical entity within 30 days or 5 half-lives, or twice the duration of the biological effect of the drug (whichever is longer) prior to the first dose of trial medication, Visit 3 (Day 1).
17. History of alcohol/drug abuse or dependence within 6 months of the trial, Screening Visit 1 (Day -45 to -14).
18. Women of childbearing potential are excluded from this trial. Women must be of non-childbearing potential [i.e. either postmenopausal or documented hysterectomy and/or bilateral oophorectomy – tubal ligation is not sufficient]. For the purposes of this trial, postmenopausal is defined as being amenorrhoeic for greater than 2 years with an appropriate clinical profile, e.g. age appropriate, history of vasomotor symptoms.

    An unwillingness of male patients to abstain from sexual intercourse with pregnant or lactating women; or an unwillingness of the patient to use a condom/spermicide in addition to having their female partner use another form of contraception such as an intrauterine device (IUD), diaphragm with spermicide, injectable progesterone, sub-dermal implants or a tubal ligation.
19. Any medical history or clinically relevant abnormality that is deemed by the principal investigator and/or medical monitor to make the patient ineligible for inclusion because of a safety concern.
20. Use of systemic corticosteroids (oral or IV) 4 weeks prior to Visit 2 (Day -14 to -1).

10.3 Method of Blinding

The placebo tablets will be manufactured to appear identical to the Losmapimod tablets. Packaging and labelling at point of supply to the patient will be blinded against the active preparation. The site pharmacy will be unblinded. Further details regarding blinding are as described in the pharmacy section of the TPM.

Recall of IMP for any reason, including a manufacturing defect, will be managed by Cambridge Clinical Trials Unit.

10.4 Emergency Unblinding

To ensure 24 hour access the services of the local pharmacy department will be used: a pharmacist on call will be available through switchboard: CUH - 01223 245 151 & RBH - 020 7352 8121.

The following questions will be asked of a physician prior to any unblinding taking place:

- Name (CI or PI or designated named person)
- Details of the AE or SAE
- Reason for needing the unblinding

Pharmacy will then follow the unblinding procedure as described in the pharmacy section of the TPM.

10.5 Participant Withdrawal Criteria and Stopping Criteria

Reasons for participant withdrawal will be recorded in the electronic Case Report Form (eCRF). Primary reasons for withdrawal may include: SUSAR, adverse event, withdrawal of consent, lost to follow-up, protocol deviation, participant non compliance, lack of efficacy, participant reached protocol-defined stopping criteria or trial closed/terminated. All the above are at the discretion of CI/PI.

In addition participants must be ≥ 70% to ≤100% compliant on taking trial medication between each on-treatment visits. Participants who fall outside this range should be re-educated on treatment compliance. In assessing compliance, it should be noted that participants may discontinue their use of trial drug due to an exacerbation. See section 10.5.3 for further information concerning participants' withdrawal due to exacerbation.

Specific withdrawal criteria relating to biochemistry, ECG abnormalities and exacerbation of COPD are listed below. Participants who are withdrawn from the trial will be replaced at the discretion of the CI/PI.

10.5.1 Biochemistry

Liver chemistry threshold stopping criteria have been designed to assure subject safety and to evaluate liver event aetiology (in alignment with the US Food and Drug Administration premarketing clinical liver safety guidance).

IMP will be stopped for a participant if any of the following liver chemistry stopping criteria is met:

1. ALT ≥ 3xULN and total bilirubin ≥ 2xULN (>35% direct bilirubin*);

   *serum bilirubin fractionation should be performed if testing is available; if unavailable, withdraw subject (if ALT ≥ 3xULN and total bilirubin ≥ 2xULN) and measure urinary bilirubin via dipstick.
2. ALT ≥ 5xULN.
3. ALT ≥ 3xULN if associated with symptoms (new or worsening) believed to be related to hepatitis (such as fatigue, nausea, vomiting, right upper quadrant pain or tenderness or jaundice) or hypersensitivity (such as fever, rash or eosinophilia).
4. ALT ≥ 3xULN persists for ≥ 4 weeks.
5. ALT ≥ 3xULN and cannot be monitored weekly for 4 weeks.

6. Participants with ALT ≥ 3xULN **and** < 5xULN **and** bilirubin < 2xULN, who do not exhibit hepatitis symptoms or rash, can continue IMP as long as they can be monitored weekly for 4 weeks. See Section 14. Details on weekly follow-up procedures for these participants.

10.5.2 ECG

Participant that meets the criteria below will be withdrawn from the trial. The QT correction formula used to determine discontinuation should be the same one used throughout the trial.

For participants with underlying bundle branch block, follow the criteria listed below:

Table 1:

Baseline QTc with bundle branch block	Discontinuation QTc with bundle branch block
<450 msec	>500 msec
450-<480 msec	≥530 msec

Change from baseline: QTc >60 msec*

*Withdrawal decisions are to be based on an average QTc value of triplicate ECGs. If an ECG demonstrates a prolonged QT interval, obtain 2 more ECGs over a brief period, and then use the averaged QTc values of the 3 ECGs to determine whether the subject should be discontinued from the trial.

10.5.3 Withdrawals due to COPD exacerbations

Participants with exacerbation of COPD may remain in the trial, and continue to take the trial medication. However if the PI or clinical team feel there is an over-riding clinical need for withdrawal (e.g. intubation) the participant will be withdrawn. Participants who are required to stop trial medication for more than 3 days must be withdrawn. The use of systemic steroids or antibiotics will be recorded at each trial visit. Patients with an exacerbation of COPD (defined as requiring systemic corticosteroids) within 4 weeks of the last treatment visit V9 will be excluded from the primary analyses.

If a participant is withdrawn due to an exacerbation, the exacerbation section and AE/SAE section of the eCRF must be completed and the participant should be followed until resolution of exacerbation.

11 Trial Treatments

11.1 Data from Non-Clinical Studies

Losmapimod is a novel p38 MAPK inhibitor which is in clinical development Phase II. For further information concerning data from Non-Clinical studies please refer to the IB.

11.2 Clinical Data

11.2.1 Efficacy

After completion of phase I trials in healthy volunteers, Losmapimod 7.5 mg BD was studied in a 12 week randomized clinical trial in COPD patients[24]. Heat shock protein[17], a downstream effector of p38 MAP kinase was significantly reduced compared with placebo after 4 and 12 weeks of therapy in the group that received losmapimod (ratio of effect of Losmapimod/placebo 0.56, 95% CI 0.43 to 0.73, p<0.001 at 4 weeks and ratio of effect of Losmapimod/placebo 0.61 95% CI 0.46 to 0.82, p<0.001 at 12 weeks). Losmapimod significantly reduced plasma fibrinogen by 11% (-0.4 g/L, placebo adjusted mean at 12 weeks 3.6 g/L, Losmapimod adjusted mean at 12 weeks 3.2 g/L; ratio of effect of Losmapimod/placebo 0.89, 95% CI 0.83 to 0.96; p=0.002). There was a trend in ratio

of effect for Losmapimod/placebo of 0.81 for IL-6 (95% CI 0.63 to 1.03; p=0.09), 0.83 for IL-8 (95% CI 0.66 to 1.04; p=0.10) and 0.76 for CRP (95% CI 0.55 to 1.06; p=0.10. The administration of Losmapimod resulted in a non-significant increase in post-bronchodilator forced vital capacity (92mL; 95% CI: -5 to 190mL; p=0.06) and non-significant reductions in both residual volume (124mL; 95% CI: -9 to 257mL; p=0.07) and thoracic gas volume (113mL, 95% CI: -20 to 246mL; p=0.09) when compared to placebo (Table 2). There was no effect on inspiratory capacity.

11.2.2 Safety and tolerability

Based on the latest data provided by GSK at the time of finalisation of this EVOLUTION protocol, since November 2011:

Table 2:

Approximately 1000 subjects have received at least 1 dose (up to 60mg single dose).
Approximately 800 subjects received 7.5mg BD (for up to 3 months).
Approximately 300 subjects have been dosed for ≥3 months.
Approximately 450 subjects are completing dosing in December 2011 in an ongoing 6-month Phase IIb COPD trial (up to 15mg BD).

This information supersedes data provided in the IB.

The longest duration of dosing in completed studies is 3 months. As above, a trial of 6 months duration completed dosing in Dec 2011 in patients with COPD, at doses of 2.5 and 7.5 mg BD for 6 months, and 7.5 mg BD for one month followed by 15 mg BD for 5 months. No safety concerns have been identified in these trials via review of blinded data in an ongoing fashion.

On-therapy AEs in repeat dose studies in patients have occurred more frequently in Losmapimod vs placebo groups (61% vs 53%), but no concerning trends have been identified with regard to any particular event. The most frequent AE was headache in Losmapimod vs placebo (13% vs 16%). Many of the events were only reported in one instance, making any distinction difficult. Pyrexia was noted more frequently in Losmapimod groups than placebo. Most of the cases were reported in two 28 day studies in neuropathic pain syndromes and many were associated with mild illnesses such as nasopharyngitis and gastroenteritis. These events were mostly classified as mild or moderate and did not result in withdrawal of trial drug.

Liver events as defined by subjects experiencing ALT ≥3x ULN have occurred with similar frequency in Losmapimod and placebo treated groups (12 (0.9%) v. 3(0.5%) respectively). Although slightly more cases of ALT elevations have occurred in Losmapimod compared to placebo, most of these cases had confounding factors that could contribute to elevation in transaminases and occurred in a recent trial completed in patients with acute coronary syndrome. All were asymptomatic outside of underlying disease states. There were 2 cases in which LFTs increased after drug had been stopped, but in one case, there was a mixed pattern of LFT increases, and in the other cases, ALT increase occurred weeks after stopping drug, which would be atypical for a drug effect, or with other cases seen with Losmapimod to date. Overall, most of the liver events seem related to confounding factors or underlying disease process, and no definitive liver

safety signal is identified. Liver events continue to monitored closely in all ongoing trials with Losmapimod.

11.2.3 Pharmacokinetics & pharmacodynamics

The predominant route of elimination of GW856553 was via urine (approximately 65% of the dose) with approximately 29% of the dose eliminated via faeces. Elimination of GW856553 was almost exclusively by metabolism with only 2% of the administered dose recovered as unchanged drug in urine and faeces. The principal route of metabolism was via hydrolysis of the cyclopropylamide group to form a pharmacologically inactive metabolite, GSK198602. Absorption of unchanged GW856553 from a solution dose was high based on the low percentage recovery of parent drug in faeces (approximately 2% of administered dose) and the high recovery of drug-related material in urine (65% of administered dose).

After single oral dose administration of 1 to 60 mg GW856553, C_{max} and area under the plasma concentration time curve $(AUC)_{(0-\infty)}$ of GW856553 increased in an approximately dose proportional manner for doses up to 20 mg and less than proportionally from 20 mg to 60 mg. The terminal half-life of GW856553 was approximately 10 hours. After single oral dose administration of GW856553, exposure (AUC) was approximately 2-fold higher for metabolite GW198602 compared to parent GW856553.

Steady-state plasma concentrations of GW856553 were achieved 2 to 3 days after initiation of repeat dosing. Slight accumulation (up to approximately 30%, on average) for AUC and C_{max} after once or twice daily dosing was observed. Consistent with single dose administration, exposure (AUC) was approximately 2-fold higher for metabolite GW198602 compared to parent GW856553.

A high fat meal increased AUC (~10%) and C_{max} (~40%) of GW856553 compared to the fasted state. Time to maximum plasma concentration (T_{max}) was delayed, on average, by 1 hour when GW856553 was administered with a high fat meal.

In general, systemic exposure appears to be similar in rheumatoid arthritis patients, COPD patients, and patients with dyslipidemia compared to healthy volunteers.

The effect of age on the pharmacokinetics of GW856553 was evaluated after single dose administration of 20 mg GW856553 to 12 elderly patients (age 65 to 75 years) and 12 young patients (age 33 to 52 years). The results indicated that exposure ($AUC_{(0-\infty)}$ and C_{max}) was similar for the two groups.

The effect of gender on the pharmacokinetics of GW856553 was informally evaluated after single dose administration of 20 mg GW856553 to 6 female and 6 male elderly patients. On average, $AUC_{(0-\infty)}$ and C_{max} were slightly increased (approximately 25% and 28%, respectively) in the female patients.

Rosuvastatin (a substrate for the organic anion transporting polypeptide 1B1 and breast cancer resistance protein transporters) $AUC_{(0\text{-}tau)}$ and C_{max} were modestly increased (30% and 25%, respectively) following administration of rosuvastatin 10 mg once daily and GW856553 15 mg once daily for 14 days compared to administration of rosuvastatin alone. These increases in rosuvastatin exposure are not considered clinically significant.

After administration of GW856553 to healthy volunteers, *ex vivo* production of LPS-stimulated TNFα and sorbitol-stimulated phosphorylated heat shock protein (pHSP)-27 in whole blood were both inhibited. The pharmacokinetic/pharmacodynamic relationships between GW856553 plasma concentration and LPS-stimulated TNFα inhibition, and

sorbitol stimulated pHSP-27 inhibition, were adequately described by sigmoid maximum effect (E_{max}) models. The estimated maximum inhibition for TNFα was 78% with an effective concentration 50% (EC_{50}) of 9.12 ng/mL. The estimated maximum inhibition for pHSP-27 was 76% with an EC_{50} of 8.45 ng/mL.
Further details may be found in Section 5.2 of the IB.

11.3 Dose Rationale

Although there are little clinical dose-response data with Losmapimod that can provide insight on an efficacious dose, in trial MKI101678, the *ex vivo* monocyte expression of TNF α (LPS treated) and sorbitol stimulated monocytes revealed pHSP27 were reduced by over 50% following 7mg Losmapimod and the inhibitions were generally higher and lasted longer following single dose of 20mg and 60mg of Losmapimod compared to 7mg.

In COPD patients (trial MKI106209) there was evidence of LPS-stimulated TNFα inhibition following repeat dosing with 7.5mg BD Losmapimod compared with placebo, when adjusted for pre-dose levels on Day 1. Results for the treatment ratios relative to placebo indicated that 7.5mg Losmapimod twice-daily demonstrated a statistically significant increase in inhibition of LPS-stimulated TNFα at 2 h post-dose on Day 1, at pre- and 2 h post-dose on Day 7, and pre-, 2 h and 6 h post-dose on Day 14 (particularly at 2 h post-dose on each of the days).

Analysis of plasma fibrinogen based on the limited data from the COPD Phase 2a trial MKI102428 showed a trend of greater reduction in fibrinogen with increase in steady state average exposure of Losmapimod in a non-linear form. Additionally, the modeling of ex vivo pHSP27 and systemic Losmapimod exposure indicated approximately 30% inhibition at trough and about 60% and inhibition at peak following 7.5mg BD dose.

The 7.5mg BD dose level yielded average (range) AUC and C_{max} values of 522 ng.hr/mL (291-933 ng.hr/mL) and 41.2 ng/mL (23.7-64.2 ng.hr/mL), respectively. These exposure values are well below those observed when subjects were given a single dose of 60mg Losmapimod (GW856553) as a micronized tablet under fed conditions (1226-2247 ng.hr/mL and 133-335 ng/mL) and this dose was found to be well-tolerated. In addition, the maximum individual AUC value is approximately 9-fold lower than the NOAEL AUC value for the 28 day rat trial and approximately 20-fold lower than the NOAEL AUC value for the 28 day monkey trial.

11.4 IMP Dosage and Administration

Losmapimod 7.5mg BD (twice daily): 1 x 7.5mg tablet each morning and each evening for 16 weeks. Placebo: one Placebo tablet to match Losmapimod each morning and each evening, for 16 weeks

Dispensing of IMP will be on V3, V5, V6 and V7. At the point of initial dispensation, the bottles will contain 70 tablets. Participants will be expected to bring their trial medication in with them every visit.

Participants will be required to swallow each tablet with approximately 240mL of water.

Table 3:

Regimen	Description
A	7.5mg Losmapimod BD
P	Placebo BD

11.4.1 Maximum dosage allowed

Participants will receive Losmapimod 7.5mg BD or Placebo BD. This makes 15mg Losmapimod the maximum daily dose allowed in this clinical trial.

11.4.2 Maximum duration of treatment of a participant

The maximum duration of treatment will be 112 days.

11.5 Descriptions: Losmapimod and matching Placebo

Losmapimod tablets (wet granulation formulation) are available as white, film coated, round, bi-convex tablets manufactured using micronised Losmapimod active substance. Tablets are available containing 7.5 mg of Losmapimod and are packed into high-density polyethylene (HDPE) bottles .

The placebo product visually matches the GW856553 Tablets 7.5 mg. The film coat used for the placebo product, Opadry white OY-S-28876, is identical to that used for the active tablets. The placebo product is manufactured using commonly used and recognised tablet excipients that are also employed in the active tablets and are packed into high-density polyethylene (HDPE) bottles.

The complete statement of the components and quantitative composition of Placebo to Match GW856553 Tablets is given in Table 4.

Table 4: Composition of Placebo to Match GW856553 Tablets

Component	Theoretical Unit Quantity (mg/tablet)	Function	Reference to Standard
Lactose Monohydrate	79.88	Diluent	Ph.Eur. or USP/NF
Microcrystalline Cellulose	60.0	Diluent	Ph.Eur. or USP/NF
Sodium Starch Glycolate	9.0	Disintegrant	Ph.Eur. or USP/NF
Magnesium Stearate	1.13	Lubricant	Ph.Eur. or USP/NF
**Core Tablet Weight**	**150.0**	-	-
Opadry White OY-S-28876(1,2)	4.5	Film coat	Supplier
Purified Water(3)	qs	Suspending Fluid	Ph.Eur. or USP
**Film Coated Tablet Weight**	**154.5**	-	-

Notes:

1. The film coat contains the polymer hypromellose Ph.Eur (E464), the colourant titanium dioxide Ph.Eur. (E171) and the plasticizer Macrogol 400 Ph.Eur.
2. Opadry white OY-S-28876 is prepared typically as a 12-13% w/w aqueous suspension. The weight of film coat applied per tablet may vary depending upon the efficiency of the process but is typically 3 % of the tablet core weight.
3. Removed during processing. Does not appear in final product ..

11.5.1 Specification for placebo tablets

Clinical trial batches for placebo tablets will meet the following specification at release and throughout the shelf-life.

Table 5: Specification of Placebo to match GW856553 tablets

Test	Acceptance Criteria
**Description**	White, round, biconvex, plain, film coated tablet

**Absence of GW856553X by HPLC1**	Not detected by HPLC analysis (less than 0.1% with respect to GW856553X)

Notes:

1. All placebo batches will comply with the test for absence of GW856553X if tested. When batches are manufactured in the same facility as the active material, the above test is performed using the method described in the IMPD for GW856553 Tablets. Where generic placebo batches to match multiple active products are made in facilities where the active products are not produced, absence of active is assured through the application of GMP and by review of batch records.

11.6 Losmapimod drug interactions

There are no clinically significant interactions with other drugs. Special precautions with BCRP substrates with a narrow therapeutic index (daunorubicin, doxorubicin, topotecan, mitoxantrone) are advised (Section 11.2.3), therefore patients on these drugs will not be permitted to participate in the trial, nor are these medications permitted during the trial. Please refer to the latest version of the IB for more information.

11.7 Handling and Storage of IMP

GSK will be providing the IMP for free of charge for the use in this trial. For further details of finished product processes and distribution to Cambridge University Hospitals NHS Foundation Trust and Royal Brompton & Harefield Foundation NHS Trust, refer to the pharmacy section of the TPM.

The IMP must be dispensed and administered according to procedures described in the TPM pharmacy section. Only participants enrolled in the trial may receive the IMP, in accordance with all applicable regulatory requirements. Only authorized site staff may supply or administer IMP. All IMP must be stored in a secure area with access limited to the investigator and authorized site staff and under physical conditions that are consistent with IMP-specific requirements.

IMP must be stored at room temperature up to 30^0C (86^0F). The expiry date is stated on the product label, for each package of IMP.

11.8 IMP Accountability

The investigator and institution, is responsible for IMP accountability, reconciliation, and record maintenance. In accordance with all applicable regulatory requirements, the investigator, or designated site staff (e.g., pharmacist or pharmacy staff, where applicable) must maintain IMP accountability records throughout the course of the trial. The responsible person(s) will document the amount of IMP received, the amount supplied and/or administered to and returned by patients. The responsible person(s) in pharmacy will destroy any returned drug once permission from the sponsor has been received.

11.9 Treatment Assignment

Participants will be randomised to the Losmapimod or Placebo regimen in a 1:1 ratio. The randomisation will be stratified by treatment centre. A delegated member of the trial team will log into the website https://prod.tenalea.net/cctu/DM/default.aspx using a secure password. They will enter the following information

- Subject Number
- Subject Initials
- Subject DoB
- Confirmation of eligibility
- Inc/Exclusion criteria confirmation

The system will provide a box number which will be used by the site pharmacy to provide the treatment allocated in a double-blinded manner.

11.10 Treatment Compliance

When participants are dosed at the trial site, they will receive IMP directly from an appropriately trained and delegated member of the clinical research team. The date and time of each dose administered in the clinic will be recorded in the source documents. The dose of IMP will be confirmed prior to dosing by a member of the trial site staff other than the person administering the IMP. A member of the clinical research team will examine each patient's mouth to ensure that the IMP was swallowed.

Participants will be required to bring back their trial medication bottles to each treatment visit. Participants remaining medication will be then counted to confirm compliance.

11.11 Concomitant Medications and Non-Drug Therapies

All concomitant medications will be recorded in the eCRF.

Participants taking systemic steroids (IV or Oral) within 4 weeks of the final treatment visit (V9) will be excluded from the primary analysis.

11.12 Treatment after EVOLUTION Trial

There will be no treatment with Losmapimod after the end of the trial. Participants enrolled into this trial will already be receiving the appropriate standard of care, and this care will be continued at the end of the trial.

11.13 Treatment of IMP Overdose

Any signs or symptoms of overdose will be treated symptomatically. No specific antidote is known and the investigator will use clinical judgment. Participants that have taken an overdose of IMP will be withdrawn from the trial.

11.14 Legal status of the drug

Losmapimod is an unlicensed investigational medicinal product currently in development by GlaxoSmithKline (GSK) for the treatment of cardiovascular disease and chronic obstructive pulmonary disease (COPD). Losmapimod has also been evaluated in patients with rheumatoid arthritis, major depressive disorder and neuropathic pain syndromes but these indications are no longer being investigated.

The trial is being carried out under a Clinical Trial Authorisation. The drug is therefore only to be used by the named investigators, for the participants specified in this protocol, and within the trial.

12 Procedures and Trial Assessments

Trial assessments and procedures will be performed by suitably qualified and delegated trial personnel as described in the TPM. Informed consent must be obtained from the participant prior to any trial-specific procedures taking place.

12.1 Trial Visits

The following sections describe in detail the procedures and investigations participants will be subjected to. Further details are available in the Trial Procedures Manual and the electronic Case Report Forms (eCRFs).

12.1.1 Timing of assessments

Please refer to Table 7 page 55 for timings of assessments

12.1.2 Screening, V1 (Day -45 to Day -14)

Participants will attend the unit for a screening visit within 45 days prior to administration of the first dose of trial medication (V3). Written informed consent must be obtained prior to performance of any protocol specific procedures. The following procedures will be performed:

- Medical history including smoking history.
- Current medication history prior to the screening visit.
- Demography (date of birth, age, gender and race).
- Physical examination including height, weight, BMI, vital signs.
- 12-lead ECG (triplicate).
- Spirometry post bronchodilator.
- Following an overnight fast, blood (approximately 55ml) and urine will be collected for clinical chemistry(including urea, creatinine, uric acid, electrolytes, liver function and glucose and HbA1c if diabetic), Full Blood Count (including haemoglobin, platelets and differential white cell count), HIV, Hepatitis B surface antigen, Hepatitis C antibody, urinalysis.
- Biomarkers.

Participants detailed cardiovascular and respiratory (and other systems) medical history will be collected, including information regarding any known allergies to drugs, use of concomitant medications, detailed history of smoking and alcohol consumption and history of blood donation and/or participation in clinical trials. The 45 day screening period may be extended if patients have an exacerbation that requires steroid or antibiotic use. This period will be extended by at least the equivalent time of steroid use added onto the 45 day period or up to the discretion of the PI where clinical judgement is required. Patients who have failed screening because of changes to trial inclusion/exclusion criteria or did not attend screening due to medical reasons (i.e. an exacerbation) and their medical status has now changed may be rescreened if they are keen to participate in the trial and happy to undergo a repeat screening, at the discretion of the PI and based on clinical judgement.

12.1.3 V2 Imaging visit - FDG-PET/CT (Day -14 to Day -1)

Between Days -14 to -1, participants will undergo a FDG-PET/CT scan. A clinical report without significant incidental findings allows the participant to be randomized into the trial, unless the findings are deemed to not require further investigation at the discretion of the PI, or after subsequent investigation before dosing with Losmapimod. Any significant findings at this visit necessitating withdrawal from the trial will be termed an Adverse Event unrelated to drug as this scan precedes drug administration. Patients who have attended the imaging visit but do not progress within 14 days onto the trial due to the necessity to investigate abnormalities on the PET-CT / MRI scan may be considered for subsequent entry onto the trial without undergoing a repeated imaging visit, if performed within a reasonable timeframe, and at the discretion of the PI and based on clinical judgement.

FDG-PET/CT imaging is expected to last for approximately 2hrs 20 minutes with a 30 minute break.

12.1.4 (Optional) V2 Imaging visit - MRI (Day -14 to Day -1)

Between Days -14 to -1, participants who are eligible for MRI scans according to local imaging centre criteria will be invited to attend an MRI scan either on the same day or

on another day according to availability. A clinical report without significant incidental findings allows the participant to be randomized into the trial, unless the findings are deemed to not require further investigation at the discretion of the PI, or after subsequent investigation before dosing with Losmapimod. Any significant findings at this visit necessitating withdrawal from the trial will be termed an Adverse Event unrelated to drug as this scan precedes drug administration.

12.1.5 Treatment Period

At each visit, procedures/assessments can take place in any order, but dosing should only occur after all assessments have been completed.

V3 (Day 1):

All assessments at this visit should be completed **prior** to participants receiving IMP.

- Concomitant medication check (confirm no systemic corticosteroids have not been used since V1).
- Vital Signs.
- Blood (55ml in total) and urine collection for clinical chemistry (including lipid profile), haematology, urinalysis.
- Blood collection for biomarkers (inclusive within the 55ml as above).
- 12 Lead ECG (triplicate).
- Adverse event assessment.
- Arterial haemodynamics: arterial stiffness, BP and HR.
- Flow mediated dilatation.
- Spirometry post bronchodilator (FEV_1 and FVC).
- SNIP (Sniff Nasal Inspiratory Pressure) measurements.
- 6 minute walk test.
- Randomisation and administration of first dose of IMP, and provision of IMP in participant packs.
- Biomarkers Fibrinogen and hsCRP (inclusive within the 55ml as above).
- Blood collection for serum store (inclusive within the 55ml as above).

V4, V5, V6, V7 (Days 14/28/56/84 (All ± 96h)):

- Concomitant medication check.
- Vital Signs.
- Blood (55ml in total) and urine collection for clinical chemistry, haematology, urinalysis.
- Biomarkers Fibrinogen and hsCRP (inclusive within the 55ml as above).
- Adverse event assessment.
- Dosing of IMP (and provision of new trial medication pack on V5 Day 28, V6 Day 56 and V7 Day 84).
- IMP tablet count.

V8 Imaging visit (Days 105-111):

- The repeat FDG-PET/CT scan can take place any day between Days 105-111.
- The repeat MRI scan (for those participants this applies to) can take place any day between Days 105-111.
- Adverse event assessment.

A clinical report will be generated for the post dose scans as part of Clinical Governance.

V9 (Days 109-112):

During any of these days (but ideally should take place on Day 112):

- Concomitant medication check.
- Vital Signs.
- Blood (55ml in total) and urine collection for clinical chemistry (including lipid profile), haematology, urinalysis.
- Biomarkers Fibrinogen and hsCRP (inclusive within the 55ml as above).
- Blood collection for serum store (inclusive within the 55ml as above).
- Adverse event assessment.
- Arterial haemodynamics: arterial stiffness, BP and HR.
- Flow mediated dilatation.
- SNIP (Sniff Nasal Inspiratory Pressure) measurements.
- 6 minute walk test.
- 12 Lead ECG (triplicate).
- Spirometry post bronchodilator.
- IMP Compliance check and final medication retrieval.

12.1.6 V10 Follow-up (Days 120 -126)

Participants will return to the unit within approximately 1-2 weeks after the last scan, and will undergo the following procedures:

- Physical examination including seated blood pressure (BP) and heart rate (HR).
- Blood (approximately 55mL) and urine will be collected for clinical chemistry, haematology, urinalysis.
- Bloods for biomarkers.
- Adverse event and concomitant medication check.

12.2 Clinical laboratory assessments

Blood samples for clinical chemistry (including liver function tests [ALT, AST, bilirubin and creatine Phosphokinase]), hematology and urine samples will be collected multiple times throughout the trial. Participants are required to fast overnight prior to these blood collections since measurement of glucose and cholesterol fractionation must occur in a fasted state. In addition to the below-mentioned haematology, clinical chemistry and urinalysis labs, additional lab studies may be performed as deemed necessary by the PI or medical monitor for the purpose of clinical evaluation or safety monitoring.

Haematology
Haemoglobin, Hematocrit, Platelets, Total White Blood cells (WBC), Neutrophils, Lymphocytes, Monocytes, Eosinophils, Basophils, and Fibrinogen.

Clinical Chemistry
Sodium, Potassium, Glucose, Creatinine, Bilirubin (total; direct and indirect will be reported if total is > 2ULN), Alanine transaminase (ALT), γ-glutamyl transferase (Gamma GT), Alkaline Phosphatase, urea uric acid, HbA1C and hsCRP.

Lipid Profile
Triglycerides, Cholesterol, High Density lipoprotein (HDL) and Low Density lipoprotein (LDL) will also be measured on V3 and V9.

Urinalysis
Approximately, 10-20ml mid-stream urine will be collected into a sterile container and will be screened by dipstick for: Protein, Ketones, Glucose, RBC, WBC.

Serum storage for up to 5 years for further analysis or trial close, whichever is sooner.

12.3 Pregnancy

The investigator will attempt to collect pregnancy information on any female partner of a male trial participant who becomes pregnant while participating in this trial. The investigator will record pregnancy information on the appropriate form and submit it to the sponsor and GSK of learning of the partner's pregnancy. The partner will also be followed to determine the outcome of the pregnancy. Information on the status of the mother and child will be forwarded to the sponsor and GSK. Generally, follow-up will be no longer than 6 to 8 weeks following the estimated delivery date. Any premature termination of the pregnancy will be reported.

12.4 Imaging

12.4.1 FDG-PET/CT

All FDG-PET/CT scans will be conducted by experienced radiographers and radiologists in the nominated scanning sites. FDG-PET/CT will be performed at the Imanova Clinical Imaging Centre, Burlington Danes Building, Imperial College on the Hammersmith Hospital site, London and Addenbrooke's PET CT Unit, Cambridge.

After 6h of fasting, providing blood glucose concentration is ≤11mmol/L trial participants will proceed to receive an FDG-PET/CT scan. This will include two CT scans of the lungs. These will be done on V2 (Day -14 to Day -1) and V8 (Days 105-111).

FDG will be administered intravenously at a dose of approximately 240 MBq.

The emission scanning will include a 60 minute dynamic acquisition from the lungs followed by aorta and the carotids. The schematic below outlines the different components and approximate timings of the FDG-PET/CT scan.

During scanning, venous blood will be drawn through an intravenous cannula inserted into the antecubital vein to measure whole blood/plasma radioactivity. This will require 12 discrete samples of approximately 5ml to be drawn, during the scan.

For each intravenous administration of ^{18}FDG the exposure (with CT) will be approximately 4 mSv due to the different scanners used at each scanning center. The total exposure of both intravenous administrations of ^{18}FDG (with both CT imaging sessions) will be approximately 22 mSv.

Please refer to the TPM for further details and specific guidelines for performing the FDG-PET/CT scans.

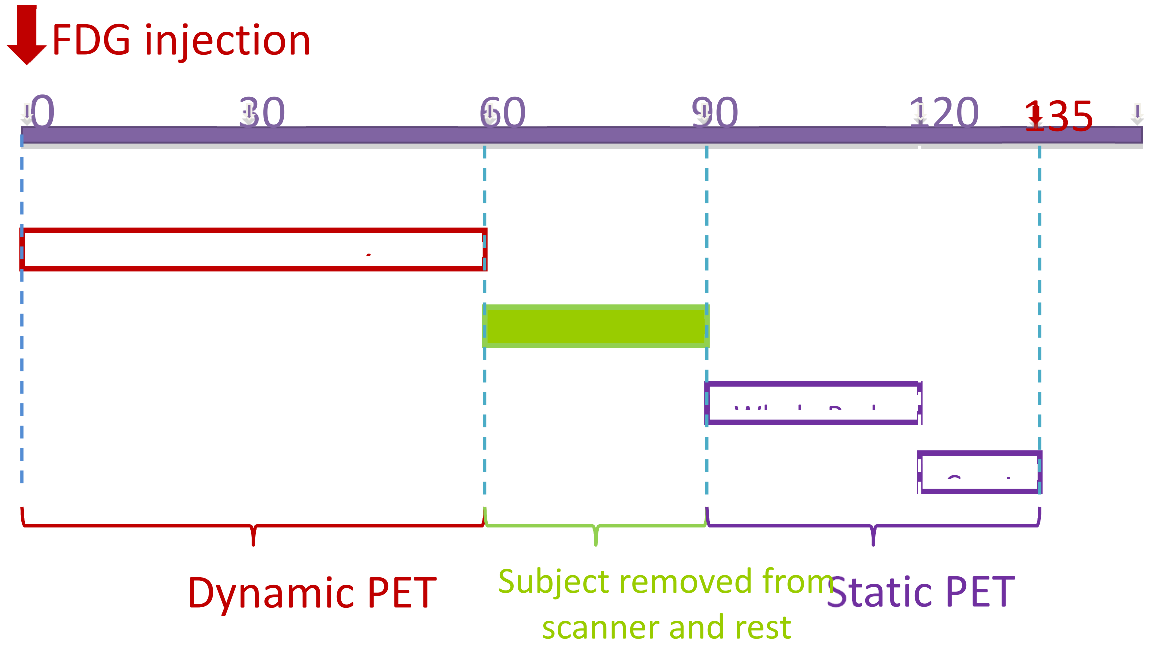

12.4.2 MRI

All MRI scans will be conducted by experienced radiographers and radiologists in the nominated scanning sites. Where possible, the same machine will be used for both MRIs on a particular participant (although the machine used may differ between participants at different scanning sites).

Contraindications to MRIs will be determined at the time of participant screening according to local clinical guidelines. Baseline MRI scanning will be performed in eligible participants between the index FDG-PET/CT scan and first drug dosing. Follow-up scans will be undertaken within a week of the final PET/CT scan at 16 weeks. Scanning duration is estimated to be no longer than 90 minutes per participant.

Participants unwilling or unsuitable for the MRI may still take part in the main study.

Detailed information on the MRI scan is provided in the TPM.

12.5 Non Imaging End Points

12.5.1 Flow mediated dilatation

Endothelial function will be assessed in the brachial artery of the non-dominant arm using the technique of flow mediated dilatation[18]. Vessel diameter will be measured, using high-resolution vascular ultrasound with a 5-20MHz linear-array transducer, continuously for 1 minute at baseline, and for a further 5 minutes following release of a cuff inflated to 200mmHg, placed distal to the ultrasound probe. After return to baseline, vessel diameter will be measured continuously for 5 minutes following administration of 25µg of sublingual glyceryl trinitrate (GTN). FMD is defined as the maximum percentage increase in vessel diameter during reactive hyperemia; GTN-mediated dilatation is defined as the maximum percentage increase in vessel diameter after sublingual GTN. This is a standard technique.

Additional details on the procedure are provided in the TPM.

12.5.2 Muscle Assessments

Inspiratory muscle strength will be measured as maximal SNIP. The largest of a minimum 5 efforts will be recorded.

12.5.3 Arterial haemodynamics

Blood pressure will be measured in the dominant arm using a validated oscillometric technique (BHS validation) following 5 minutes of seated rest. Measurements will be made in triplicate and the average of the last 2 will be used for analysis. Additional readings will be made if there is an error or an erroneous reading. Heart rate will also be recorded. Following this, a high-fidelity micro manometer will be used to obtain accurate readings of the peripheral radial pressure waveforms for arterial haemodynamics in the seated position by flattening, but not occluding, the radial artery of the dominant arm using gentle pressure. Data will be collected to produce a central pressure waveform. Augmentation represents the difference between the second and first systolic peaks of the central pressure waveform. AIx is defined as the augmentation expressed as a percentage of the overall pulse pressure. Heart rate will also be reported, MAP and central pressure.

Following 15 minutes supine rest BP will be recorded as detailed above. Aortic Pulse wave velocity (PWV) will then be determined between the carotid and femoral arteries using a non-invasive pressure sensor and ECG gating, and reported together with heart rate and calculated MAP. Additional readings will be made if there is an error or erroneous reading.

More detailed information on how to perform this procedure is listed in the TPM.

12.6 Six minute walk test

A 6 minute walk test will be conducted in all participants to assess exercise tolerance, according to ATS guidelines. This is a simple, self-paced exercise test, where participants are asked to walk at their own pace for 6 minutes, including breaks if necessary. The distance covered on a flat, hard surface (ie. walking track) with minimal blind turns or obstacles will be recorded as the 6 minute walking distance.

12.7 Blood Sampling

Blood samples will be collected on all visits. Blood samples will be collected pre-dose, following an overnight fast.

Blood samples will be collected to analyze established inflammatory biomarkers, at the cellular (total blood leukocytes, blood neutrophils) and protein level (e.g. hsCRP, plasma fibrinogen) but not limited to these.

After completion of the clinical trial, investigations may be performed on samples collected during the course of the trial to detect factors or profiles that correlate with other measures of response to treatment with Losmapimod. The resultant data may also be of application for medically related conditions.

- Performance of these investigations may be conditional on the results of the clinical trial principally, but not exclusively, on the primary measures of the clinical trial outcome and samples may be selected for analysis on the basis of the trial outcome.

Comparative examination of pre-dosing profiles of participants may uncover known or novel candidate biomarkers/profiles which could be used to predict response to treatment

with Losmapimod or provide new insights into medically related conditions. Comparative examination of post-dosing profiles in conjunction with pre-dosing profiles may yield known and novel candidate biomarkers/profiles and new insights which relate to the action of Losmapimod.

All other samples will be retained for a maximum of 5 years after the last participant completes the trial.

Please refer to the TPM for sample processing information.

12.8 Trial restrictions

12.8.1 Meal and Dietary Restrictions

Participants will be asked to abstain from ingestion of alcohol and caffeine 12 hours prior to the first dose of trial medication and until collection of the final blood sample at each individual trial visit. Participants will be required to fast overnight prior to each clinical chemistry and haematology blood sample collection. Participants undergoing FDG-PET/CT and ± MRI scanning will be required to fast for 6 hours prior to scanning. Previous studies have not identified a major effect of food in the systemic exposure of Losmapimod; therefore, there are no food restrictions other than the ones indicated above.

12.8.2 Other Restrictions

Participants should abstain from strenuous physical activity for 72 hours prior to each blood collection for clinical laboratory tests. Participants who are smokers will be unable to smoke for the duration of each trial visit. Participants will be required to fast for 6 hours before scanning and restrict exercise for 24 hours before FDG-PET/CT and ± MRI scanning.

12.8.3 Contraception

Men must agree not to have sexual intercourse with a woman who is pregnant or breastfeeding a baby from the time of first dosing until the end of the trial without adequate contraception. They also must avoid fathering children during the trial and follow up period. This means that they must not have sex, or if they have sexual intercourse with a female of child-bearing potential, they must use a condom with spermicide, and preferably the female should use some other form of birth control, such as a diaphragm with spermicide, an intrauterine device (IUD), birth control pills, injectable progesterone, sub dermal implants or a tubal ligation for the duration of the trial and follow up period. Women must be of non-childbearing potential [i.e. either postmenopausal or documented hysterectomy – tubal ligation is not sufficient]. For the purposes of this trial, post menopausal is defined as being amenorrhoeic for greater than 2 years with an appropriate clinical profile, e.g. age appropriate, history of vasomotor symptoms. Surgical sterility will be defined as females who have had a hysterectomy and/or bilateral oophorectomy.

12.9 Safety

Clinical safety data is a secondary endpoint of this Phase 2 trial. The safety and tolerability of protocol-specified treatments will be assessed by physical exam findings, 12-lead ECGs recordings, vital sign (blood pressure and heart rate) measurements, clinical laboratory tests, clinical monitoring /observation and spontaneous and elicited adverse event reporting.

13 Assessment of Safety

13.1 Safety Reporting Definitions

The investigator or trial staff are responsible for detecting, documenting and reporting events that meet the definition of an AE or SAE.

AEs will be recorded from the start of IMP dosing V3 (Day1), V4, V5, V6, V7 (Days 14/28/56/84 (All ± 96h)), V8 (Days 105-111), V9 (Days 109-112) and until the follow-up V10 (Days 120-126).

SAEs will be collected over the same time period as stated above for AEs. However, any SAEs assessed as related to trial participation (e.g. trial treatment, protocol-mandated procedures, invasive tests, or change in existing therapy) or related to a GSK product will be recorded from the time a participant consents to participate in the trial up to and including any follow-up visit. All SAEs will be recorded and reported to sponsor/CI and GSK within 24 hours of awareness, as indicated in TPM.

Investigators are not obligated to actively seek AEs or SAEs in former trial participants. However, if the investigator learns of any SAE, including a death, at any time after a participant has been discharged from the trial, and he/she considers the event reasonably related to the IMP or trial participation, the investigator would promptly notify sponsor/CI and GSK.

13.1.1 Definition of Adverse Events

An AE is any untoward medical occurrence in a participant or clinical investigation subject, temporally associated with the use of a medicinal product, whether or not considered related to the medicinal product.

Note: An AE can therefore be any unfavorable and unintended sign (including an abnormal laboratory finding), symptom, or disease (new or exacerbated) temporally associated with the use of a medicinal product.

Events meeting the definition of an AE include:

- Any abnormal laboratory test results (haematology, clinical chemistry, or urinalysis) or other safety assessments (e.g., ECGs, radiological scans, vital signs measurements), including those that worsen from baseline, and felt to be clinically significant in the medical and scientific judgment of the investigator.
- Exacerbation of a chronic or intermittent pre-existing condition including either an increase in frequency and/or intensity of the condition.
- New conditions detected or diagnosed after IMP administration even though it may have been present prior to the start of the trial.
- Signs, symptoms, or the clinical sequelae of a suspected interaction.
- Signs, symptoms, or the clinical sequelae of a suspected overdose of either IMP or a concomitant medication (overdose per se will not be reported as an AE/SAE unless this is an intentional overdose taken with possible suicidal/self-harming intent. This should be reported regardless of sequelae.).
- "Lack of efficacy" or "failure of expected pharmacological action" per se will not be reported as an AE or SAE. However, the signs and symptoms and/or clinical sequelae resulting from lack of efficacy will be reported if they fulfill the definition of an AE or SAE.

Events that do not meet the definition of an AE include:

- Any clinically significant abnormal laboratory findings or other abnormal safety assessments that are associated with the underlying disease, unless judged by the investigator to be more severe than expected for the subject's condition.
- The disease/disorder being studied, or expected progression, signs, or symptoms of the disease/disorder being studied, unless more severe than expected for the participants condition.
- Medical or surgical procedure (e.g., endoscopy, appendectomy); the condition that leads to the procedure is an AE.
- Situations where an untoward medical occurrence did not occur (social and/or convenience admission to a hospital).

Anticipated day-to-day fluctuations of pre-existing disease(s) or condition(s) present or detected at the start of the trial that do not worsen.

13.1.2 Definition of Serious Adverse Events

If an event is not an AE as defined in Section 13.1.1, then it cannot be an SAE even if serious conditions are met (e.g., hospitalization for signs/symptoms of the disease under trial, death due to progression of disease, etc).

An SAE is any untoward medical occurrence that:

- Results in death
- Is life-threatening

NOTE: The term 'life-threatening' in the definition of 'serious' refers to an event in which the participant was at risk of death at the time of the event. It does not refer to an event, which hypothetically might have caused death, if it were more severe.

- Requires hospitalisation or prolongation of existing hospitalisation

NOTE: In general, hospitalisation signifies that the participant has been detained (usually involving at least an overnight stay) at the hospital or emergency ward for observation and/or treatment that would not have been appropriate in the physician's office or out-patient setting. Complications that occur during hospitalization are AEs. If a complication prolongs hospitalization or fulfills any other serious criteria, the event is serious. When in doubt as to whether "hospitalisation" occurred or was necessary, the AE should be considered serious.

NOTE: Hospitalisation for elective treatment of a pre-existing condition that did not worsen from baseline V3 is not considered an AE.

- Results in disability/incapacity, or

  NOTE: The term disability means a substantial disruption of a person's ability to conduct normal life functions. This definition is not intended to include experiences of relatively minor medical significance such as uncomplicated headache, nausea, vomiting, diarrhea, influenza, and accidental trauma (e.g. sprained ankle) which may interfere or prevent everyday life functions but do not constitute a substantial disruption.

- Is a congenital anomaly/birth defect

- Is associated with liver injury and impaired liver function defined as:
- ALT ≥ 3xULN and total bilirubin* ≥ 2xULN (>35% direct), **or** INR > 1.5** in lieu of bilirubin ≥ 2 x ULNALT ≥ 3xULN and INR** > 1.5.

  * Serum bilirubin fractionation should be performed if testing is available; if unavailable, measure urinary bilirubin via dipstick. If fractionation is unavailable and ALT ≥ 3xULN and total bilirubin ≥ 2xULN, then the event is still to be reported as an SAE.

  ** INR testing not required per protocol and the threshold value does not apply to participants receiving anticoagulants. If INR measurement is obtained, the value is to be recorded on the SAE form.

Medical or scientific judgment should be exercised in deciding whether reporting is appropriate in other situations, such as important medical events that may not be immediately life-threatening or result in death or hospitalization but may jeopardize the participant or may require medical or surgical intervention to prevent one of the other outcomes listed in the above definition. These should also be considered serious. Examples of such events are invasive or malignant cancers, intensive treatment in an emergency room or at home for allergic bronchospasm, blood dyscrasias or convulsions that do not result in hospitalization, or development of drug dependency or drug abuse.

13.2 Evaluating AEs and SAEs

13.2.1 Assessment of Intensity

The investigator will make an assessment of intensity for each AE and SAE reported during the trial and will assign it to one of the following categories:

Mild: An event that is easily tolerated by the participant, causing minimal discomfort and not interfering with everyday activities.

Moderate: An event that is sufficiently discomforting to interfere with normal everyday activities.

Severe: An event that prevents normal everyday activities.

An AE that is assessed as severe will not be confused with an SAE. Severity is a category utilized for rating the intensity of an event; and both AEs and SAEs can be assessed as severe. An event is defined as 'serious' when it meets at least one of the pre-defined outcomes as described in the definition of an SAE.

13.2.2 Assessment of Causality

The investigator is obligated to assess the relationship between IMP and the occurrence of each AE/SAE. A "reasonable possibility" is meant to convey that there are facts/evidence or arguments to suggest a causal relationship, rather than a relationship cannot be ruled out. The investigator will use clinical judgment to determine the relationship. Alternative causes, such as natural history of the underlying diseases, concomitant therapy, other risk factors, and the temporal relationship of the event to the IMP will be considered and investigated. The investigator will also consult the IB and/or SmPC, for marketed products, in the determination of their assessment.
For each AE/SAE the investigator must document in the medical notes that they have reviewed the AE/SAE and has provided an assessment of causality.

Causality assessment may be categorized as follows:

Definitely: A causal relationship is clinically/biologically certain. This is therefore an Adverse Reaction

Probable: A causal relationship is clinically / biologically highly plausible and there is a plausible time sequence between onset of the AE and administration of the investigational medicinal product and there is a reasonable response on withdrawal. This is therefore an Adverse Reaction

Possible: A causal relationship is clinically / biologically plausible and there is a plausible time sequence between onset of the AE and administration of the investigational medicinal product. This is therefore an Adverse Reaction

Unlikely: A causal relation is improbable and another documented cause of the AE is most plausible. This is therefore an Adverse Event.

Unrelated: A causal relationship can be definitely excluded and another documented cause of the AE is most plausible. This is therefore an Adverse Event.

13.2.3 Expectedness

An adverse event that has been described as having a causal association with the medicine in the applicable product information (typically the developmental core safety information, DCSI in the IB) in the IB is considered to be an expected event.

When the adverse event is not consistent with the applicable product information this event is considered unexpected.

13.3 Expected Serious Adverse Events

Please refer to the safety profile as detailed in the IB, but so far no causal relationship has been suggested in any of the reported AEs or SAEs.

13.4 Recording and evaluation of AEs and SAEs

Individual adverse events should be evaluated by the investigator. This includes the evaluation of its seriousness, causality and any relationship between the investigational medicinal product(s) and/or concomitant therapy and the adverse event.

When an AE/SAE occurs, it is the responsibility of the investigator to review all documentation (e.g., hospital progress notes, laboratory, and diagnostics reports) relative to the event. The investigator will then record all relevant information regarding an AE/SAE in the appropriate data collection tool.

The investigator will attempt to establish a diagnosis of the event based on signs, symptoms, and/or other clinical information. In such cases, the diagnosis will be documented as the AE/SAE and not the individual signs/symptoms.

Each Principal Investigator needs to record all adverse events and report these to the Chief Investigator. The Chief Investigator is responsible assessing all AE's and SAEs for expectedness and the prompt notification of all SAEs to the Sponsor. The sponsor has to keep detailed records of all SAEs reported to them by the trial team. For further information please refer to SOP002 pharmacovigilance for investigator teams. This will also be in the TPM.

Table 6:

Action	Timeline
Reporting SAE to Sponsor by sending it to CCTU	24 hours of Investigator awareness
Reporting SUSAR Sponsor by sending it to CCTU	24 hours of Investigator awareness
Reporting SUSAR to REC & MHRA	7 days – fatal and life threatening<br>15 days – all others
Returning query responses to CCTU	As advised in query, depending on the nature of query and urgency

13.5 Reporting of Suspected Unexpected Serious Adverse Reactions (SUSARs)

All suspected adverse reactions related to an investigational medicinal product (the tested IMP and placebo) which occur in this trial, and that are both unexpected and serious (SUSARs) are subject to expedited reporting.

13.5.1 Who should report and whom to report to?

The sponsor delegates the responsibility of notification to the MHRA, REC and any other investigators to the Chief Investigator. As appropriate for investigational therapies in Phase II development, safety and tolerability of Losmapimod will be assessed throughout this trial and reported to the sponsor and GSK.

The Chief Investigator should report all the relevant safety information previously described, to the sponsor, MHRA and to the Ethics Committee concerned. The Chief Investigator shall inform all investigators concerned of relevant information about SUSARs that could adversely affect the safety of patients.

13.5.2 When to report?

13.5.2.1 Fatal or life-threatening SUSARs

The MHRA, Research Ethics Committee, Sponsor and GSK should be notified as soon as possible (ideally between 24 – 72 hours of knowledge) but no later than **7 calendar days** after the trial team and Sponsor has first knowledge of the minimum criteria for expedited reporting.

In each case relevant follow-up information should be sought and a report completed as soon as possible. It should be communicated to the MHRA, Ethics Committee and Sponsor within an additional **8 calendar days**.

13.5.2.2 Non fatal and non life-threatening SUSARs

All other SUSARs and safety issues must be reported to the MHRA and Ethics Committee as soon as possible within 7 calendar days but no later than **15 calendar days** after first knowledge of the minimum criteria for expedited reporting. Further relevant follow-up information should be provided as soon as possible.

13.5.3 How to report?

13.5.3.1 Minimum criteria for initial expedited reporting of SUSARs

Information on the final description and evaluation of an adverse reaction report may not be available within the required time frames for reporting. For regulatory purposes, initial expedited reports should be submitted within the time limits as soon as the minimum following criteria are met:
a) a suspected investigational medicinal product,
b) an identifiable participant (e.g. trial subject code number),

c) an adverse event assessed as serious and unexpected, and for which there is a reasonable suspected causal relationship,
d) an identifiable reporting source,

and, when available and applicable:
- an unique clinical trial identification (EudraCT number)
- an unique case identification (i.e. sponsor's case identification number).

13.5.3.2 Follow-up reports of SUSARs

In case of incomplete information at the time of initial reporting, all the appropriate information for an adequate analysis of causality should be actively sought from the reporter or other available sources. Further available relevant information should be reported as follow-up reports.

In certain cases, it may be appropriate to conduct follow-up of the long-term outcome of a particular reaction.

13.5.3.3 Format of the SUSARs reports

Electronic reporting is the expected method for expedited reporting of SUSARs to the MHRA. The format and content as defined by the MHRA should be adhered to, please refer to the TPM for further information.

14 Toxicity – Emergency Procedures

No serious toxicity has been seen with Losmapimod. The manufacturer is keen to record all "Sentinel Events" pertaining to liver dysfunction. Therefore, the trial incorporates all safety procedures, in terms of clinical management, as recommended by GlaxoSmithKline. These are detailed below.

In the event of a SAE relating to liver test dysfunction then the following provides guidance on appropriate clinical investigation, from GSK and the regulatory authorities. Should an SAE arise participants will be managed through established NHS clinical pathways as clinically indicated. The trial team and clinical team will, to the best of their ability, undertake the investigations listed below and report these to Sponsor /GSK as safety-related data, but follow local governance procedures at all times.

- Immediately withdraw the participant from IMP
- Notify the medical monitor (as listed on Page 1) within 24 hours of learning of the abnormality to confirm the subject's IMP cessation and follow-up.
- Complete the "Safety Follow-Up Procedures" listed below.
- Complete the liver event eCRF. If the event also meets the criteria of an SAE (see Section 13.1.2), the SAE data collection tool will be completed separately with the relevant details.
- Upon completion of the safety follow-up withdraw the participant from the trial unless further safety follow up is required.

14.1.1 Safety Follow-Up Procedures for participants with ALT ≥ 3xULN and bilirubin ≥ 2xULN (>35% direct)

This event is considered an SAE (see Section 13.1.2). Serum bilirubin fractionation should be performed if testing is available. If fractionation is unavailable, urinary

bilirubin is to be measured via dipstick (a measurement of direct bilirubin, which would suggest liver injury).

- Make every reasonable attempt to have the participants return to the clinic (within 24 hours) for repeat liver chemistries, additional testing and to be monitored closely (with specialist or hepatology consultation recommended).
- Monitor participants twice weekly until liver chemistries (ALT, AST, alkaline phosphatase, bilirubin) resolve, stabilize or return to within baseline values.

14.1.2 Safety Follow-Up Procedures for participants with ALT ≥ 5xULN or ALT ≥ 3xULN who have hepatitis symptoms or rash, can't be monitored for 4 weeks or have elevations that persist ≥ 4 weeks [Stopping Criteria section 10.5]:

- Make every reasonable attempt to have the subject return to the clinic within 24-72 hours for repeat liver chemistries and additional testing.
- Monitor participants weekly until liver chemistries (ALT, AST, alkaline phosphatase, bilirubin) resolve, stabilize or return to within baseline values.

14.1.3 Safety Follow-Up Procedures for participants with ALT ≥ 3xULN and < 5xULN and bilirubin < 2xULN, who do not exhibit hepatitis symptoms or rash:

- Notify the medical monitor within 24 hours of learning of the abnormality to discuss participant safety.
- Participant can continue IMP if liver chemistries (ALT, AST, alkaline phosphatase, bilirubin) can be monitored weekly for up to 4 weeks.
- If at any point these participants meet the liver chemistry stopping criteria (outlined in Section 10.5), immediately withdraw IMP, perform additional testing and continue safety follow-up until liver chemistries resolve, stabilize or return to baseline values.
- After 4 weeks of monitoring, if ALT < 3xULN and bilirubin < 2xULN, participants must be monitored twice monthly until liver chemistries normalize or return to within baseline values.

14.1.4 Recommended additional Follow-Up Procedures for participants who meet *any* of the stopping criteria:

- Viral hepatitis serology including:
- Hepatitis A IgM antibody;
- Hepatitis B surface antigen and Hepatitis B Core Antibody (IgM);
- Hepatitis C RNA;
- Cytomegalovirus IgM antibody;
- Epstein-Barr viral capsid antigen IgM antibody (or if unavailable, obtain heterophile antibody or monospot testing);
- Hepatitis E IgM antibody.
- record the time of the last dose
- Serum creatine phosphokinase (CPK) and lactate dehydrogenase (LDH).
- Fractionated bilirubin, if total bilirubin ≥ 2xULN.
- Assess eosinophilia

- Record the appearance or worsening of clinical symptoms of hepatitis (fatigue, nausea, vomiting, right upper quadrant pain or tenderness, fever, rash or eosinophilia) as relevant on the AE CRF
- Record use of concomitant medications, paracetamol, herbal remedies, other over the counter medications, or putative hepatotoxins on the Concomitant Medications CRF.
- Record alcohol use on the Liver Events eCRF.

The following are required for participants with ALT ≥ 3xULN **and** bilirubin ≥ 2xULN (>35% direct) but are optional for other abnormal liver chemistries:

- Anti-nuclear antibody, anti-smooth muscle antibody, Type 1 anti-liver kidney microsomal antibodies and quantitative total immunoglobulin G (IgG or gamma globulins).
- Serum paracetamol adduct HPLC assay (quantifies potential paracetamol contribution to liver injury in participants with definite or likely paracetamol use in the preceding week[25]).
- Liver imaging (ultrasound, magnetic resonance, or computerized tomography) to evaluate liver disease.
- The Liver Imaging and/or Liver Biopsy eCRFs are also to be completed if these tests are performed.

The procedures listed below are to be followed if a subject has ALT, bilirubin and/or INR elevations that meet the definition of an SAE (as defined in Section 13.1.2):

- Notify the medical monitor within 24 hours of learning of the abnormality to confirm follow-up.
- Complete the liver event eCRFs.
- Upon completion of the safety follow-up withdraw the subject from the trial unless further safety follow up is required.
- Make every reasonable attempt to have participants return to the clinic within 24 hours for repeat liver chemistries, additional testing, and close monitoring (with specialist or hepatology consultation recommended).
- Monitor participants twice weekly until liver chemistries (ALT, AST, alkaline phosphatase, bilirubin) resolve, stabilize or return to within baseline values.
- Obtain viral hepatitis serology including:
- Hepatitis A IgM antibody.
- Hepatitis B surface antigen and Hepatitis B Core Antibody (IgM).
- Hepatitis C RNA.
- Cytomegalovirus IgM antibody.
- Epstein-Barr viral capsid antigen IgM antibody (or if unavailable, obtain heterophile antibody or monospot testing).
- Hepatitis E IgM antibody.
- Serum creatine phosphokinase (CPK) and lactate dehydrogenase (LDH).
- Assess eosinophilia
- Record the appearance or worsening of clinical symptoms of hepatitis (fatigue, nausea, vomiting, right upper quadrant pain or tenderness, fever, rash or eosinophilia) on the AE CRF.

- Record use of concomitant medications, paracetamol, herbal remedies, other over the counter medications, or putative hepatotoxins on the Concomitant Medications CRF.
- Record alcohol use on the Liver Events CRF.
- Anti-nuclear antibody, anti-smooth muscle antibody, Type 1 anti-liver kidney microsomal antibodies and quantitative total immunoglobulin G (IgG or gamma globulins).
- Serum paracetamol adduct HPLC assay (quantifies potential paracetamol contribution to liver injury in patients with definite or likely paracetamol use in the preceding week[21]
- Liver imaging (ultrasound, magnetic resonance, or computerized tomography) to evaluate liver disease.

The Liver Imaging and/or Liver Biopsy eCRFs are also to be completed if these tests are performed

15 Evaluation of Results

Primary:

Change in vascular inflammation (using derivatives of Standard Uptake Value (SUV) and Tissue to Background Ratio (TBR) in the carotid and aortic arteries of patients following 16 weeks of treatment.

Change in flow mediated dilatation following 16 weeks treatment.

Change in atheromatous plaque and vessel wall characteristic using MRI and/or FDG-PET/CT, following 16 weeks of treatment. MRI analysis will be dependent on a sufficient number of datasets since this was an optional sub-study. In the case of insufficient datasets the MRI primary endpoint will not be analysed and reported in the primary analysis of this clinical trial, and may be included as an exploratory analysis. A minimum of 85% of paired MRI scans (i.e. pre and post dose in the same patient) of all the MRI scans performed in this sub-study will be deemed to be sufficient. The scans will need to be of high image quality, and without any technical issues which preclude analysis (e.g. patient movement, ECG gating etc.).

Secondary:

Change in lung inflammation assessed by FDG-PET/CT, following 16 weeks of treatment.

Change in visceral and subcutaneous fat inflammation, and serum/plasma measures of systemic inflammation, following 16 weeks of treatment.

Change in arterial stiffness measures (aortic pulse wave velocity and augmentation index), following 16 weeks of treatment.

Change in baseline corrected blood concentration of biomarkers.

Change in spirometry (FEV_1, FVC, FEV_1/FVC ratio), sniff nasal inspiratory pressures and 6MWT following 16 weeks of treatment.

Change in body composition as measured by body mass index (BMI) and free fat mass index (FFMI), following a 16 week treatment.

Safety and tolerability parameters, including physical examination, blood pressure, heart rate, 12-lead electrocardiogram (ECGs) recordings, clinical laboratory tests, lung function, and adverse event reporting

16 Statistics

16.1 Statistical methods

The primary focus of the trial is the quantification of vascular inflammation using the PET/CT images. From such images a Standard Uptake Value (SUV) and a Tissue to Blood Ratio (TBR) (both measures of FDG uptake) will be calculated using dedicated software.

Subsequent manipulation and interpretation of the TBR/SUV data is not well established and will be open to revisions based on the data observed. It is hypothesized that Losmapimod acts only on tissue that is already inflamed, hence the interpretation aims to focus only on inflamed tissue, but the identification and tracking of such tissue between the observations is subjective.

The identification will be performed by trained trial staff using Osirix software to trace the border of each vessel/section within each slice. See the Imaging section within the TPM for further operational details. From each slice and vessel/section identified, a maximum and mean SUV and TBR value will be calculated over the associated region of the slice by the software. These values will be referred to as max-SUV, mean-SUV, max-TBR and mean-TBR respectively.

Within each participant visit the vessel with the highest number of slices with max-TBR>1.6 will be identified within each participant at the initial visit and subsequently followed. This will be termed the "index vessel".

Within each vessel and participant visit the following variables will be derived from either for each vessel and also for the index vessel:

1. Maximum value over all slices for max-SUV, mean-SUV, max-TBR & mean-TBR
2. Mean value over all slices for max-SUV, mean-SUV, max-TBR & mean-TBR: The whole vessel means.
3. Incidence of slices with max-TBR>1.6
4. Incidence of slices with mean-TBR>1.6
5. The mean of the max-TBR value over the five contiguous slices centred on the slice with the highest max-TBR in the vessel: the most diseased segment mean of max-TBR.

For all derived variables considered, summary statistics of the post-treatment values and change from baseline will be presented broken down by treatment group. Summary statistics for continuous endpoints are n (number of observations), mean, sd, median, minimum, maximum. Summary statistics for incidence or categorical variables are percentage, count and n – p% (x/n).

Formal ANCOVA analysis will be performed on the change from baseline, with fixed effects for the treatment and adjustment for baseline value and treatment centre. Estimates of the treatment effect, (Losmapimod – Placebo) will be provided with 95% confidence intervals and p-values. All estimated regression parameters will be reported in a similar fashion. Comparisons of proportions will be handled using a similar approach based on logistic regression to estimate odd ratios.

As an exploratory trial the focus will be estimation and interpretation of treatment effects and not on formal hypothesis tests. As such no adjustment for the extensive multiple testing will be done.

Secondary endpoints will have similar summary measures reported broken down by time-point and treatment group. Endpoints that are repeatedly observed in the intermediate visits during treatment will be summarised graphically. Regression analysis may be performed.

A detailed statistical analysis plan will be finalised prior to data base lock, although it is anticipated that this will be the first stage in reporting and subsequent post-hoc analyses will be performed and documented as being post-hoc.

Figure 1 illustrates some of these derived variables.

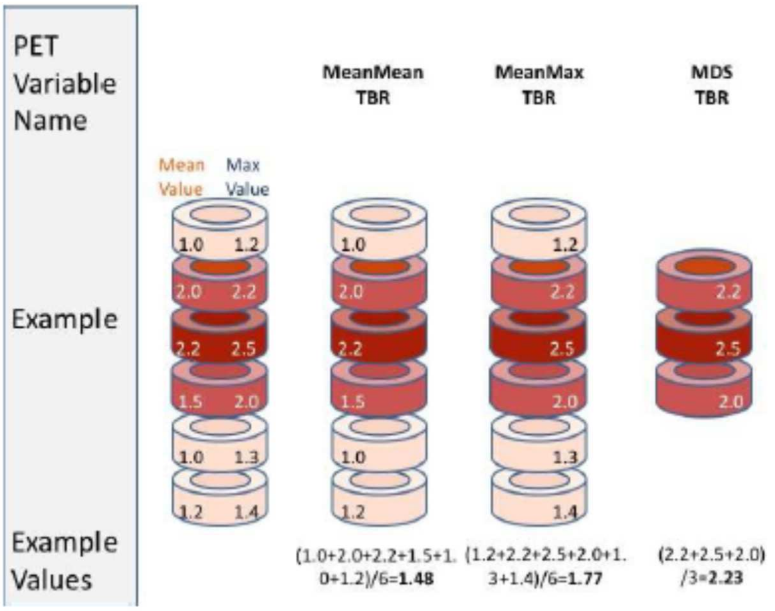

Figure 1: Stylised diagram of an artery to demonstrate the various PET indices that are derived from the regions of interest (ROI) placed on the vessel. A circular ROI is drawn to encompass the vessel wall on each contiguous axial segment. Each axial segment then provides two numbers: a mean and a maximal value for the FDG uptake within that segment, expressed as the TBR. The MeanMean TBR is the averaged Mean TBR values from each artery. The MeanMax TBR is the averaged Max TBR values from each artery. The most diseased segment (MDS) is centred on the darkest red segment, defined as having the highest maximal TBR value in the entire artery. When averaged with the segments immediately above and below it, these three segments yield the MDS. Example values and their derivations are provided in the lowest row

16.2 Analysis Populations

Inflammation Population: All participants who provide baseline (V3) and post-dose (V9) scans but excluding those taking systemic steroids within 4 weeks prior to V9 will be included in the primary statistical analysis population. This population will be used in the evaluation of inflammation data.

Safety Population: All participants who receive at least one dose of trial drug will be included in the safety population. Randomized participants will only be excluded if there is clear documented evidence of failure to take any trial medication. Participants in the

Safety Population will be classified according to the treatment received. All safety summaries will be based on the Safety Population.

Pharmacodynamic Population: All participants who provide baseline and V8 pharmacodynamic (PD) data will be included in the PD population. This population will be used in the evaluation of PD.

MRI Population: All participants who provide baseline V3 and V9 MRI data will be included in the MRI population. This population will be used in the evaluation of MRI data.

16.3 Derived and Transformed Data

Details of the derived endpoints will be provided in the Reporting and Analysis Plan. As this is an exploratory trial, any data transformations may be data-driven; i.e., based on a preliminary examination of the data, but wherever possible, will be detailed in the Reporting and Analysis Plan.

16.4 Number of Participants to be enrolled

A sufficient number of participants will be enrolled so that approximately 60 participants who fulfil the primary statistical anaylsis population as described in Section 16.2 (approximately 30 participants per arm) complete the trial. Sample size is based in part on feasibility.

As an exploratory trial a formal power calculation is inappropriate. The analysis will focus on estimating the differences between treatments and will provide 95% confidence intervals of the form $x \pm y$, where x is the estimate and y is the confidence interval half-width. A smaller value of the half-width is desirable as it gives greater precision to the trial's conclusions. The value of the half-width depends, on the SD of the endpoint under consideration, the number of participants and an element of chance variation.

Based on the results of a previous GSK trial, PM1111138 "A double-blind, placebo-controlled, parallel group trial to evaluate the effects of two regimens of GW856553, over a period of 3 month, on in-vivo macrophage activity, as assessed by FDG-PET/CT imaging, in the carotid arteries and aorta of patients with established atherosclerosis" a SD of around 0.3 is estimated in many of the endpoints on the TBR scale that were considered. Under this assumption, with 30 participants per arm, there is a chance of 90% of achieving a half-width of 0.17 or less.

Such precision will be meaningful for drawing conclusion. Moreover a sample size of 30 participants per arm will provide enough data to be able to check assumptions behind the statistical analyses.

16.5 Criteria for the termination of the trial

Any new evidence from previous or current studies that Losmapimod may have serious detrimental effects in the trial population, in the opinion of the CI, the trial will be discontinued. Another criterion will be failure to recruit sufficient participants to complete, in the opinion of the CI, analysis of the data collected.

16.6 Procedure to account for missing or spurious data

The primary analyses will be performed on the pharmacodynamic population. Formal regression analysis will be based on the subset of participants who have all data points observed: complete cases. This assumes that the reason for any missing data is independent and unrelated to the data values, either observed or unobserved. The level of missing data will be documented and sensitivity analysis considered if the incidence of missing data in the primary endpoint is greater than 20%.

All summary statistics will report both the population size and the number of non-missing observations. Summary statistics will ignore any missing data and thus make the same assumptions as described above.

16.7 Definition of the end of the trial

The end of trial definition will be 3 months after last participant last visit.

17 Data handling and record keeping

17.1 eCRF

All data will be transferred into a electronic Case Report Form (eCRF) which will be anonymised. All trial data in the eCRF must be extracted from and be consistent with the relevant source documents. The eCRFs must be completed, dated and signed by the investigator or designee in a timely manner. It remains the responsibility of the investigator for the timing, completeness and accuracy of the eCRF pages. The eCRF will be accessible to trial coordinators, data managers and the investigators as required.

The investigator will also supply the trial coordinator with any required, additional, background information from the medical records as required.

The investigators must ensure that the eCRFs and other trial related documentation is sent to the trial coordinator containing no participant identifiable data.

17.2 Source Data

To enable peer review, monitoring, audit and/or inspection the investigator must agree to keep records of all enrolled participants (sufficient information to link records e.g., CRFs, hospital records and samples), all original signed informed consent forms and copies of the CRF pages.

Source data includes but is not limited to:
- Informed Consent Form
- Medical Records
- ECG Print outs
- Scan images
- On-line test results systems
- Prescriptions

17.3 Data Protection

All investigators and trial site staff involved in this trial must comply with the requirements of the Data Protection Act 1998 and Trust Policy with regards to the collection, storage, processing and disclosure of personal information and will uphold the Act's core principles.

18 Trial Steering and Data Monitoring Committee

The Trial Steering Committee (TSC) is responsible for the day to day running of the trial and related activities. The composition of the TSC is stated on page 3 of the protocol. The TSC also provides overall supervision for the trial, to ensure that it is conducted in accordance with the protocol and GCP and to provide advice through its independent chairman (Professor Peter Calverley, pmacal@liv.ac.uk). The committee will aim to convene at regular intervals to review the data and discuss if the trial is on course to meet the sample size requirements. The committee will appoint a DMC and a DMC charter will be prepared prior to any DMC meetings including an interim analysis plan, although this may evolve over the course of the

trial. The DMC will be composed of persons not involved in the direct running, management or final analysis of the trial. The DMC will examine the recruitment rates, safety profile, and efficacy and mechanistic endpoints. The time scale and funding of the trial is limited and the ability of the trial to deliver data of sufficient scientific value will be considered. Adaptations to the trial may include but are not limited to:

- Revisions to the sample size, including early trial cessation
- Revisions to the randomisation ratio to reflect different variability of the primary endpoint in the treatment groups

The DMC will provide recommendations to the TSC. The TSC will retain ultimate responsibility to choose whether or not to follow the recommendation of the DMC.

19 Ethical & Regulatory considerations

19.1 Consent

The Informed Consent form must be approved by the REC and must be in compliance with ICH GCP, local regulatory requirements and legal requirements. The investigator must ensure that each trial participant is fully informed about the nature and objectives of the trial and possible risks associated with their participation.

The investigator will obtain written informed consent from each participant before any trial-specific procedure is performed. The informed consent form used for this trial and any change made during the course of this trial, must be prospectively approved by the REC. The investigator will retain the original of each participants signed informed consent form.

Should a participant require a verbal translation of the trial documentation by a locally approved interpreter/translator, it is the responsibility of the individual investigator to use locally approved translators.

19.2 Ethical committee review

Before the start of the trial or implementation of any amendment, Ethical and Regulatory approval of the trial protocol, protocol amendments, informed consent forms and other relevant documents e.g., advertisements and GP information letters if applicable will be obtained. All correspondence with the REC will be retained in the Trial Master File and the Investigator Site File.

Annual reports will be submitted to the REC in accordance with national requirements.

19.3 Regulatory Compliance

The trial will not commence until a Clinical Trial Authorisation (CTA) is obtained from the MHRA. The protocol and trial conduct will comply with the Medicines for Human Use (Clinical Trials) Regulations 2004 and any relevant amendments.

Periodic regulatory reports will be submitted to the MHRA and other EU regulatory authorities in accordance with national requirements. These will be produced and provided to the Sponsor by GSK.

19.4 Protocol Amendments

Protocol amendments must be reviewed and authorisation received from the Sponsor for all proposed amendments prior to submission to the REC and/or MHRA.

The only circumstance in which an amendment may be implemented prior to REC and/or MHRA approval is where the change is necessary to eliminate apparent, immediate risks to the patients (Urgent Safety Measures). In the case, accrual of new patients will be halted until the REC and/or MHRA approval has been obtained.

The trials co-ordinator will be responsible for the running the trial, they will prepare all amendments arrange for sponsor authorisation and make the submission.

19.5 Declaration of Helsinki and ICH Good Clinical Practice

The trial will be performed in accordance with the spirit and the letter of the declaration of Helsinki, the ICH Good Clinical Practice Guidelines, the protocol and applicable local regulatory requirements and laws.

19.6 GCP Training

All trial staff must hold evidence of appropriate GCP training or undergo GCP training prior to undertaking any responsibilities on this trial. This training should be updated every 2 years or in accordance with your Trust's policy.

20 Sponsorship, Financial and Insurance

The trial sponsor is Cambridge University Hospitals NHS Foundation Trust and the University of Cambridge. Funding has been awarded by the MRC-Technology Strategy Board (9157-61188). The award is for the period of 2 years. The grant was awarded on the basis of 3 workpackages being conducted – one) observational and two) CTIMPs. This trial, An **Ev**aluation **o**f **L**osmapimod in patients with Chronic Obstructive P**u**lmonary Disease (COPD) with sys**t**emic inflammat**ion** stratified using fibrinogen (EVOLUTION) is the second workpackage funded from this grant and is a CTIMP.

Cambridge University Hospitals NHS Foundation Trust, as a member of the NHS Clinical Negligence Scheme for Trusts, will accept full financial liability for harm caused to participants in the clinical trial caused through the negligence of its employees and honorary contract holders. There are no specific arrangements for compensation should a participant be harmed through participation in the trial, but no-one has acted negligently.

The University of Cambridge will arrange insurance for negligent harm caused as a result of protocol design and for non-negligent harm arising thorough participation in the clinical trial.

21 Monitoring, Audit & Inspection

The investigator must make all trial documentation and related records available should an MHRA Inspection occur. Should a monitoring visit or audit be requested, the investigator must make the trial documentation and source data available to the Sponsor's representative. All patient data must be handled and treated confidentially.

Monitoring will be conducted by the Clinical Trial Monitors at Cambridge Clinical Trials Unit.

22 Protocol Compliance and Breaches of GCP

The investigator must not implement any deviation from the protocol without formal written agreement from the Sponsor and Chief Investigator. If this necessitates a subsequent protocol amendment, or halt to the trial this should be submitted to the REC, MHRA & Research & Development Department for review and approval if appropriate.

Potential/suspected serious breach of GCP must be reported immediately to the Sponsor.

23 Publications policy

Ownership of the data arising from this trial resides with the trial team. On completion of the trial the data will be analysed and tabulated and a Clinical Trial Report prepared.

The investigators intend to publish the findings of the trial as conference abstract(s) and in peer- reviewed journals.

24 References

1. Mannino, D.M. & Braman, S. The epidemiology and economics of chronic obstructive pulmonary disease. *Proc Am Thorac Soc* 4, 502-506 (2007).

2. Jemal, A., Ward, E., Hao, Y. & Thun, M. Trends in the leading causes of death in the United States, 1970-2002. *JAMA* 294, 1255-1259 (2005).

3. European Medicines Agency. Guideline on clinical investigation of medicinal products in the treatment of Chronic Obstructive Pulmonary Disease (COPD). Vol. 2011 (2010).

4. Center for Drug Evaluation and Research (CDER) Food and Drug Administration. Guidance for Industry: Chronic Obstructive Pulmonary Disease: Developing Drugs

5. Agusti, A., et al. Characterisation of COPD heterogeneity in the ECLIPSE cohort. Respir Res 11, 122 (2010).

6. Sin, D.D., et al. Inhaled corticosteroids and mortality in chronic obstructive pulmonary disease. Thorax 60, 992-997 (2005).

7. Seymour, J.M. et al. Outpatient pulmonary rehabilitation following acute exacerbations of COPD. Thorax. 65, 423-428 (2010).

8. Garcia-Aymerich, J., et al. Lung function impairment, COPD hospitalisations and subsequent mortality. Thorax 66, 585-590 (2011).

9. Sabit, R., et al. Arterial Stiffness and Osteoporosis in Chronic Obstructive Pulmonary Disease. Am J Respir Crit Care Med 175, 1259-1265 (2007).

10. Stary HC, et al. A definition of advanced types of atherosclerotic lesions and a histological classification of atherosclerosis. A report from the Committee on Vascular Lesions of the Council on Arteriosclerosis, American Heart Association. Circulation. 92, 1355-1374 (1995)

11. Jaffer FA, Libby, Peter.; Weissleder, Ralph. Molecular Imaging of Cardiovascular Disease. Circulation. 116, 1052-1061 (2007).

12. Rudd JHF, Imaging atherosclerotic plaque inflammation with 18FDG-PET. Circulation. 105, 2708-11 (2002).

13. Coulson, JM, et al. Excessive aortic inflammation in chronic obstructive pulmonary disease: an 18F-FDG PET pilot study. J Nucl Med. 51, 1357-60 (2010).

14. Yun M, 18F-FDG PET in characterizing adrenal lesions detected on CT or MRI. J Nucl Med 2001 December;42(12):1795-9.

15. Tawakol A, Noninvasive in vivo measurement of vascular inflammation with F-18 fluorodeoxyglucose positron emission tomography. J Nucl Cardiol 2005 May;12(3):294-301.

16. Rudd JH, Imaging atherosclerotic plaque inflammation with [18F]-fluorodeoxyglucose positron emission tomography. Circulation 2002 June 11;105(23):2708-11.

17. Kwee RM, Potential of integrated [18F] fluorodeoxyglucose positron-emission tomography/CT in identifying vulnerable carotid plaques. AJNR Am J Neuroradiol 2011 May;32(5):950-4.

18. Tahara N, Vascular inflammation evaluated by [18F]-fluorodeoxyglucose positron emission tomography is associated with the metabolic syndrome. J Am Coll Cardiol 2007 April 10;49(14):1533-9.

19. Tawakol A, In vivo 18F-fluorodeoxyglucose positron emission tomography imaging provides a noninvasive measure of carotid plaque inflammation in patients. J Am Coll Cardiol 2006 November 7;48(9):1818-24.

20. Newsholme P, Metabolism of glucose, glutamine, long-chain fatty acids and ketone bodies by murine macrophages. Biochem J 1986 October 1;239(1):121-5.

21. Deichen JT, Uptake of [18F]fluorodeoxyglucose in human monocyte-macrophages in vitro. Eur J Nucl Med Mol Imaging 2003 February;30(2):267-73.

22. Tahara N, Simvastatin attenuates plaque inflammation: evaluation by FDG-PET. JACC. 48, 1825-31 (2006).

23. Folco EJ, Hypoxia but not inflammation augments glucose uptake in human macrophages: Implications for imaging atherosclerosis with 18fluorine-labeled 2-deoxy-D-glucose positron emission tomography. J Am Coll Cardiol 2011 August 2;58(6):603-14.

24. Sluimer JC, Hypoxia-inducible transcription factor, and macrophages in human atherosclerotic plaques are correlated with intraplaque angiogenesis. J Am Coll Cardiol 2008 April 1;51(13):1258-65.

25. James LP, Pharmacokinetics of acetaminophen-protein adducts in adults with acetaminophen overdose and acute liver failure. Drug Metab Dispos 2009 August;37(8):1779-84.

25 Appendices

25.1 Appendix 1 - Trial Management / Responsibilities

25.1.1 Data management

The Senior Data Manager employed by the Cambridge Clinical Trials Unit will be responsible for the design and implementation of the clinical trial database as well as the management of the data within the database. This shall be done via the MACRO database system using the eCRF remote data capture system, data is expected to be entered within 2 weeks of a participants visit date. The Senior Data Manager will issue and respond to queries to sites via the electronic system. These must be responded to by the local trial team within one month of receipt.

They shall also be responsible for any regular and ad-hoc data requests for example, data for DSURS and TSC meetings.

25.1.2 Preparation and submission of DSUR/Annual Progress Reports

The clinical trials coordinator will be responsible for the day to day running of the trial and submitting Annual Progress Reports. GSK will prepare all DSURs for submission. Copies will be filed in the TMF and provided to the sponsor for review, prior to submission.

25.1.3 Trial documentation & archiving

It is the responsibility of the PI at both sites to maintain the ISF. It will be the responsibility of the CTC to provide this information for filing.

All essential source and trial documentation will be securely archived after the last analysis of the trial data has been completed and the Final Trial Report has been submitted to the relevant authorities. Archiving must be provided for at least 5 years or the length of time specified by current, applicable legislation, whichever is the longer.

The Investigator must not destroy any document or records associated with the trial without written approval from the Sponsor. Each site is responsible for their own archiving arrangements and costs involved.

25.1 Time and Events Table (Table 7)

Assessments		Treatment Days								
	Screening **V1**	Day -14 to -1 Imaging Visit **V2**	Day 1 **V3**	Day 14 (Days 10-18) **V4**	Day 28 (Days 24-32) **V5**	Day 56 (Days 52-60) **V6**	Day 84 (Days 80-88) **V7**	Day 107 (Days 105-111) **Imaging Visit V8**	Day 112 (Days 109-112) **V9**	Day 120 – 126 Follow-up **V10**
Attend Unit	*	*	*	*	*	*	*	*	*	*
Medical History	*									
Demography	*									
Physical Examination	*									*
Standard supine 12-lead ECG (triplicate)	*		*						*	
Blood (55ml)Haematology (FBC, LFT etc…) Chemistry (Urea, Uric acid etc…), urinalysis	*		*	*	*	*	*		*	*
Hep B, Hep C and HIV Test, HbA1C	*									
Biomarkers (Plasma Fibrinogen, hsCRP)	*		*	*	*	*	*		*	*
Bloods For Imaging (Glucose & Radiation Levels)		*						*		
Lipid Profile, Serum Store			*						*	
FDG-PET/CT		*						*		
MRI (Optional)		*						*		
Adverse event check			*	*	*	*	*		*	*
Concomitant medication check			*	*	*	*	*		*	*
Arterial haemodynamics			*						*	
Flow mediated dilatation			*						*	
Muscle assessment (SNIP)			*						*	
Spirometry	*		*						*	
6 Minute walk test			*						*	
Randomisation			*							
Vital Signs	*		*	*	*	*	*		*	*
Dispensing of IMP			*		*	*	*			
