# Supplementary material for: The p38 mitogen activated protein kinase inhibitor losmapimod in chronic obstructive pulmonary disease patients with systemic inflammation, stratified by fibrinogen: A randomised double-blind placebo-controlled trial
Source: PLoS One. 2018 Mar 22;13(3):e0194197. doi: 10.1371/journal.pone.0194197 (PMC5863984; doi:10.1371/journal.pone.0194197)
Supplement: S1 Methods — (DOCX) [file pone.0194197.s004.docx]

**S1 Methods**

**Imaging Protocols**

A General Electric Lightspeed VCT (Milwaukee, Wisconsin) scanner was used at Cambridge and a Siemens Biograph (Munich, Germany) scanner at London. As different scanners are used at the respective sites for the trial, there are some differences in imaging protocols. However reconstruction protocols aim to minimise variability and provide quality, comparable image data for analysis.

**Positron Emission Tomography (PET) and Computed Tomography (CT):**

Subjects are required to fast for 6 hours prior to the scan and to avoid any strenuous exercise in the preceding 24 hours to limit muscle uptake of tracer. Any metal objects are removed prior to scanning and only clothing without metallic fastenings are allowed during imaging. Blood glucose levels must be less than 11mmol/L to proceed with the scan.

A dose of approximately 240Mbq ^18^F-FDG is injected, followed by 10mls flush of normal saline. After 90 minutes post injection (optimum time to allow distribution of tracer in circulation and uptake in vessel wall), a non-contrast CT scan for the aorta scan is performed. (Cambridge: 40mA, 140 kV, pitch 1.375, London: 27 mA, 130 kV, pitch 1.5) from arch to bifurcation, followed by a PET acquisition (3x10 minute bed positions in 3D mode).

Carotid artery imaging is next undertaken at approximately two hours after FDG injection. The head and neck are placed in a holder and a non-contrast CT scan of the neck is performed. (Cambridge: 40mA, 140kV, pitch 1.375, London: 30mA, 130 kV, 1.5 pitch). This is immediately followed by a single bed PET scan acquired in 3D mode for 15 minutes, where the external auditory meatus is the upper anatomical landmark of the scan. The CT scans are used for attenuation correction and anatomical co-registration. The PET list mode data for the lung scan is reconstructed to give dynamic frames for kinetic analysis and a static quality control image (45-60mins post-injection) according to specific reconstruction protocols for each scanner. PET data for the aorta and carotids is reconstructed using the default 3D iterative algorithm on each scanner. All scans are reported by consultant radiologists and any unusual diagnostic finding.

**Assessments for primary endpoint**

A further primary endpoint of the EVOLUTION trial was to evaluate aortic wall properties using magnetic resonance imaging (MRI). However, it was not possible to include or report this co-primary endpoint because of an insufficient number of complete datasets to enable analysis. As aortic MRI was an optional measurement in the study, the protocol required 85% of paired MRI scans (i.e. pre and post dose in the same patient) to be undertaken to allow analysis as a primary variable. This was not met due to patient refusal, patients not completing the study, and data acquisition problems.
