# Supplementary material for: The p38 mitogen activated protein kinase inhibitor losmapimod in chronic obstructive pulmonary disease patients with systemic inflammation, stratified by fibrinogen: A randomised double-blind placebo-controlled trial
Source: PLoS One. 2018 Mar 22;13(3):e0194197. doi: 10.1371/journal.pone.0194197 (PMC5863984; doi:10.1371/journal.pone.0194197)
Supplement: S1 Table — (DOCX) [file pone.0194197.s005.docx]

| A participant will be eligible for inclusion only if all of the following criteria apply: |
| --- |
| - Male/female aged 50-85 years of age inclusive at screening, weighing ≥ 45 kg & BMI ≤35 kg/m^2^. |
| - A clinical diagnosis of COPD with GOLD Stages 1, 2, 3, or 4, or GOLD-U. |
| - FEV_1_/FVC <0.7 post bronchodilator |
| - Participant is a smoker or an ex-smoker with a smoking history of at least 10 pack years (1 pack year = 20 cigarettes smoked per day for 1 year or equivalent). |
| - Baseline plasma fibrinogen value of >2.8 g/L (Klauss method)*. |
| - ALT < 2xULN at screening; alkaline phosphatase and bilirubin ≤ 1.5xULN at screening (isolated bilirubin >1.5xULN is acceptable if bilirubin is fractionated and direct bilirubin <35%). |
| - Subjects must have a QTc<450 msec on screening (V1) ECG (using average value of triplicate ECGs).  For patients with complete Right bundle branch block, the QTc must be <480msec on Screening V1 ECG.  Patients with other ECG findings will be excluded if warranted at the discretion of the CI/PI.  QTc readings will be QTcF. |
| - If participants consent to optional MRI, they must fulfil local imaging centre requirements.   **Trial Amendment to Inclusion Criteria**  * The plasma fibrinogen stratification threshold for entry to the trial was originally set at >3.5 g/L, since this threshold is associated with increased all-cause mortality and exacerbations in COPD patients [1]. However, due to slow recruitment (and the majority of screen failures for the trial were due to fibrinogen-see Table E3), the threshold was lower to 2.8 g/L, which is associated with increased risk of hospitalisation in the general population, and poor lung function [2]. |

References

1. Mannino D, Tal-Singer R, Lomas D, Vestbo J, Barr G, Tetzlaff K, et al. Plasma Fibrinogen as a Biomarker for Mortality and Hospitalized Exacerbations in People with COPD. J COPD F. 2014;2(1):23–34.

2. Dahl M, Tybjærg-Hansen A, Vestbo J, Lange P, Nordestgaard BG. Elevated plasma fibrinogen associated with reduced pulmonary function and increased risk of chronic obstructive pulmonary disease. Am J Respir Crit Care Med. 2001 Sep 15;164(6):1008–11.
