# Supplementary material for: The p38 mitogen activated protein kinase inhibitor losmapimod in chronic obstructive pulmonary disease patients with systemic inflammation, stratified by fibrinogen: A randomised double-blind placebo-controlled trial
Source: PLoS One. 2018 Mar 22;13(3):e0194197. doi: 10.1371/journal.pone.0194197 (PMC5863984; doi:10.1371/journal.pone.0194197)
Supplement: S2 Table — (DOCX) [file pone.0194197.s006.docx]

| The presence of any of the following will preclude patient inclusion: |
| --- |
| - Inability to provide Informed Consent. |
| - A cardiovascular event in the last 6 months |
| - Patients on daunorubicin, doxorubicin, topotecan, mitoxantrone. |
| - Previous lung reduction surgery. |
| - Known clinically significant pulmonary diagnoses in which inflammation is thought to play a role including diagnosis of bronchiectasis, sarcoidosis, lung fibrosis, interstitial lung disease, or α1-antitrypsin deficiency. |
| - Known hepatitis B or C |
| - Active or chronic liver disease, or known hepatic or biliary abnormalities |
| - Known chronic infections such as HIV or known active tuberculosis. |
| - Diagnosis of rheumatoid arthritis, connective tissue disorders and other conditions known to be associated with active chronic inflammation (e.g. Inflammatory Bowel Disease). |
| - Insulin controlled Type 1 or Type 2 diabetics. |
| - Diabetics on oral hypoglycaemics/diet with HbA1c (DCCT) > 8% (OR HbA1c (IFCC) > 64 mmol/mol), at screening. [note: fasting glucose to be checked again at first FDG-PET/CT scan, and if glucose > 11mmol/L at that visit, patients will be excluded from trial] |
| - Participation in research trial in the last 3 years which involved exposure to significant ionising radiation (i.e. cumulative research radiation dose >5 mSv) |
| - History of malignancy within the past 5 years (with the exception of localized carcinoma of the skin that has been resected for cure). |
| - Previous exposure to Losmapimod. |
| - Subjects who have donated more than 500 mL of blood within 2 months prior to the trial medication administration, Visit 3 |
| - Participation in a clinical trial where the patient has received a drug or new chemical entity within 30 days or 5 half-lives, or twice the duration of the biological effect of the drug (whichever is longer) prior to the first dose of trial medication. |
| - History of alcohol/drug abuse or dependence within the past 6 months |
| - Women of childbearing potential |
| - An unwillingness of male patients to abstain from sexual intercourse with pregnant or lactating women; or an unwillingness of the patient to use appropriate contraception |
| - Any medical history or clinically relevant abnormality that is deemed by the principal investigator and/or medical monitor to make the patient ineligible for inclusion because of a safety concern |
| - Use of systemic corticosteroids (oral or IV) prior to visit 2 |
