# Supplementary material for: The p38 mitogen activated protein kinase inhibitor losmapimod in chronic obstructive pulmonary disease patients with systemic inflammation, stratified by fibrinogen: A randomised double-blind placebo-controlled trial
Source: PLoS One. 2018 Mar 22;13(3):e0194197. doi: 10.1371/journal.pone.0194197 (PMC5863984; doi:10.1371/journal.pone.0194197)
Supplement: S3 Table — (DOCX) [file pone.0194197.s007.docx]

| **Not meeting inclusion/exclusion criteria** | **Number (%)**  **Total =69** |
| --- | --- |
| Plasma fibrinogen threshold | 40 (58%) |
| Spirometry | 6 (9%) |
| Medical reasons | 10 (15%) |
| ECG criteria | 3 (4%) |
| Body mass index | 2 (3%) |
| Glucose/Hb1ac | 1 (1%) |
| Failed two or more inclusion/exclusion criteria | 7 (10%) |
