# Supplementary material for: The p38 mitogen activated protein kinase inhibitor losmapimod in chronic obstructive pulmonary disease patients with systemic inflammation, stratified by fibrinogen: A randomised double-blind placebo-controlled trial
Source: PLoS One. 2018 Mar 22;13(3):e0194197. doi: 10.1371/journal.pone.0194197 (PMC5863984; doi:10.1371/journal.pone.0194197)
Supplement: S4 Table — (DOCX) [file pone.0194197.s008.docx]

| **Covariate** | **Estimate** | **Confidence Intervals** | **P-value** |
| --- | --- | --- | --- |
| **Index vessel TBR** | | | |
| Treatment effect | -0.05 | -0.17, 0.07 | 0.42 |
| Baseline TBR | -0.41 | -0.6, -0.18 | <0.001 |
| Site | -.04 | -0.08, 0.17 | 0.48 |
| **Aortic arch TBR** | | | |
| Treatment effect | -0.12 | -0.22, -0.03 | 0.01 |
| Baseline TBR | -0.39 | -0.59, -0.19 | <0.001 |
| Site | -0.01 | -0.11, 0.08 | 0.78 |
| **Ascending aorta TBR** | | | |
| Treatment effect | -0.04 | -0.15, 0.06 | 0.43 |
| Baseline | -0.40 | -0.62, -0.18 | <0.001 |
| Site | 0.03 | -0.08, 0.13 | 0.60 |
| **Descending aorta TBR** | | | |
| Treatment effect | -0.04 | -0.14, 0.07 | 0.49 |
| Baseline | -0.56 | -0.13, 0.08 | <0.001 |
| Site | -0.02 | -0.13, 0.08 | 0.68 |
| **Abdominal aorta TBR** | | | |
| Treatment effect | -0.06 | -0.16, 0.03 | 0.20 |
| Baseline TBR | -0.34 | -0.55, -0.12 | 0.002 |
| Site | 0.02 | -0.07, 0.11 | 0.68 |
| **Right carotid TBR** | | | |
| Treatment effect | -0.05 | -0.13, 0.04 | 0.29 |
| Baseline TBR | -0.26 | -0.52, 0.0007 | 0.05 |
| Site | -0.06 | -0.16, 0.05 | 031 |
| **Left carotid TBR** | | | |
| Treatment effect | -0.06 | -0.19, 0.07 | 0.33 |
| Baseline TBR | -0.44 | -0.78. -0.10 | 0.01 |
| Site | 0.08 | -0.06, 0.23 | 0.25 |
| **Flow mediated dilatation** | | | |
| Treatment effect | 0.4 | -1.66, 2.48 | 0.70 |
| Baseline FMD % | -0.72 | -1.18, -0.27 | 0.003 |
| Site | 1.74 | -0.77, 4.21 | 0.16 |
| Pre-test mean diameter | -0.75 | -2.11, 0.61 | 0.27 |
| **Glyceryl Trinitrate** | | | |
| Treatment effect | 3.25 | 0.41, 6.1 | 0.03 |
| Baseline GTN % | -0.81 | -1.21, -0.42 | <0.001 |
| Site | -0.4 | -3.26, 2,44 | 0.77 |
| Pre-test mean diameter | -2.71 | -4.58, -0.83 | 0.006 |

TBR=tissue-to-blood ratio. FMD=Flow mediated dilatation, GTN=Glyceryl trinitrate.

Estimate=coefficient of variable in each model. Baseline value and site included in each model. For FMD and GTN models, pre-test mean diameter of the brachial artery also included.
