# Supplementary material for: The p38 mitogen activated protein kinase inhibitor losmapimod in chronic obstructive pulmonary disease patients with systemic inflammation, stratified by fibrinogen: A randomised double-blind placebo-controlled trial
Source: PLoS One. 2018 Mar 22;13(3):e0194197. doi: 10.1371/journal.pone.0194197 (PMC5863984; doi:10.1371/journal.pone.0194197)
Supplement: S5 Table — (DOCX) [file pone.0194197.s009.docx]

|  | **Losmapimod**  **n=36** | **Placebo**  **n=37** |
| --- | --- | --- |
| **Total adverse events** | **n=85** | **n=79** |
| **Respiratory n (%)** | **34 (40%)** | **30 (38%)** |
| Dyspnoea | 6 | 2 |
| Cough | 5 | 6 |
| Cold or Flu symptoms | 3 | 9 |
| Wheeze | 1 | 1 |
| COPD exacerbation | 19 | 12 |
| **Gastrointestinal n (%)** | **12 (14%)** | **10 (13%)** |
| Abdominal pain | 5 | 1 |
| Bloating or indigestion | 2 | 1 |
| Constipation | 1 | 1 |
| Diarrhoea | 2 | 1 |
| Increased stool frequency | 0 | 1 |
| Gastroenteritis | 0 | 1 |
| Nausea or vomiting | 1 | 3 |
| PR bleed | 0 | 1 |
| Elective Surgical procedure | 1 | 0 |
| **Cardiovascular n (%)** | **1 (1%)** | **2 (3%)** |
| Atrial flutter | 0 | 1 |
| Swollen ankles | 1 | 1 |
| **Musculoskeletal n (%)** | **4 (5%)** | **7 (9%)** |
| Musculoskeletal pain or swelling | 4 | 5 |
| **CNS and Opthamological n (%)** | **6 (7%)** | **16 (20%)** |
| Dry or sore eye | 1 | 2 |
| Elective eye surgical procedure | 0 | 1 |
| Dizziness or light headed | 1 | 3 |
| Headache | 4 | 9 |
| **Skin and soft tissue n (%)** | **3 (4%)** | **3 (4%)** |
| Cellulitis | 0 | 1 |
| Basal cell carcinoma | 0 | 1 |
| Dry skin | 2 | 0 |
| Rash | 1 | 1 |
| **Renal and Urological n (%)** | **7 (8%)** | **1 (1%)** |
| Proteinuria | 1 | 0 |
| Micturition difficultly | 1 | 1 |
| Urinary tract infection | 5 | 0 |
| **Dental and ENT n (%)** | **14 (16%)** | **5 (5%)** |
| Dry mouth | 2 | 0 |
| Mouth ulcers, or gingivitis or toothache | 3 | 1 |
| Jaw pain | 0 | 1 |
| Nasal congestion, or polyps, or nose bleed | 4 | 1 |
| Sinusitis | 1 | 1 |
| Sore throat | 4 | 0 |
| Hayfever | 0 | 1 |
| **Systemic n (%)** | **3 (4%)** | **2 (3%)** |
| Fever | 1 | 0 |
| Loss of appetite or thirst | 2 | 0 |
| Tiredness | 0 | 2 |
| **Abnormal laboratory result n (%)** | **1 (1%)** | **3 (4%)** |
| Hyponatraemia | 0 | 1 |
| Elevated CRP | 0 | 1 |
| Liver function test abnormality | 1 | 1 |
| **Serious adverse events n** | 10 | 1 |
| Admission to hospital with COPD exacerbation or pneumonia n (%) | 6 (60%) | 1 (100%) |
