# Supplementary figures and images for: The p38 mitogen activated protein kinase inhibitor losmapimod in chronic obstructive pulmonary disease patients with systemic inflammation, stratified by fibrinogen: A randomised double-blind placebo-controlled trial
Source: PLoS One. 2018 Mar 22;13(3):e0194197. doi: 10.1371/journal.pone.0194197 (PMC5863984; doi:10.1371/journal.pone.0194197)

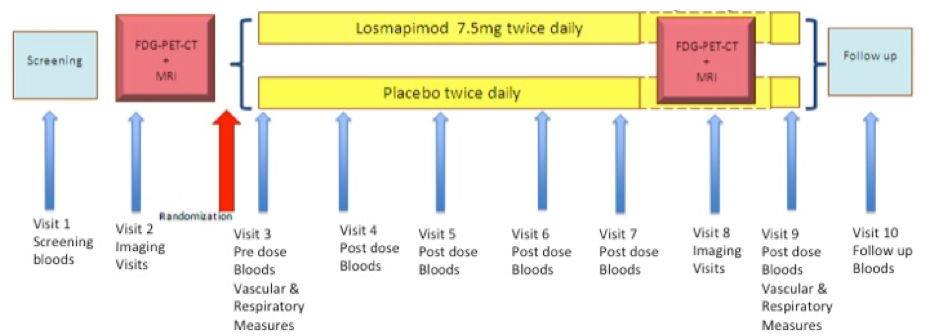

Supplement: S1 Fig — (TIFF) [file pone.0194197.s010.tiff]
